# Asia–Pacific clinical practice guidelines on the management of hepatocellular carcinoma: a 2017 update

**DOI:** 10.1007/s12072-017-9799-9

**Published:** 2017-06-15

**Authors:** Masao Omata, Ann-Lii Cheng, Norihiro Kokudo, Masatoshi Kudo, Jeong Min Lee, Jidong Jia, Ryosuke Tateishi, Kwang-Hyub Han, Yoghesh K. Chawla, Shuichiro Shiina, Wasim Jafri, Diana Alcantara Payawal, Takamasa Ohki, Sadahisa Ogasawara, Pei-Jer Chen, Cosmas Rinaldi A. Lesmana, Laurentius A. Lesmana, Rino A. Gani, Shuntaro Obi, A. Kadir Dokmeci, Shiv Kumar Sarin

**Affiliations:** 1 Department of Gastroenterology, Yamanashi Prefectural Central Hospital, Kofu-city, Yamanashi Japan; 2 The University of Tokyo, Tokyo, Japan; 3 Department of Oncology and Internal Medicine, National Taiwan University Hospital, National Taiwan University Cancer Center and Graduate Institute of Oncology, National Taiwan University, Taipei, Taiwan; 4 Hepato-Biliary-Pancreatic Surgery Division and Artificial Organ and Transplantation Division, Department of Surgery, Graduate School of Medicine, The University of Tokyo, Tokyo, Japan; 5 Department of Gastroenterology and Hepatology, Kindai University School of Medicine, Osaka-Sayama, Osaka Japan; 6 Department of Radiology and Institute of Radiation Medicine, Seoul National University College of Medicine, Seoul, Republic of Korea; 7 Beijing Key Laboratory of Translational Medicine on Cirrhosis, National Clinical Research Center for Digestive Diseases, Liver Research Center, Beijing Friendship Hospital, Capital Medical University, Beijing, China; 8 Department of Gastroenterology, Graduate School of Medicine, The University of Tokyo, Tokyo, Japan; 9 Department of Internal Medicine, Yonsei University College of Medicine, Seoul, Republic of Korea; 10 Department of Hepatology, Postgraduate Institute of Medical Education and Research, Chandigarh, India; 11 Department of Gastroenterology, Juntendo University, Tokyo, Japan; 12 Department of Medicine, Aga Khan University and Hospital, Karachi, Pakistan; 13 Department of Hepatology, Cardinal Santos Medical Center, Manila, Philippines; 14 Department of Gastroenterology, Mitsui Memorial Hospital, Tokyo, Japan; 15 Department of Gastroenterology and Nephrology, Graduate School of Medicine, Chiba University, Chiba, Japan; 16 Department of Internal Medicine, National Taiwan University Hospital, Taipei, Taiwan; 17 Digestive Disease and GI Oncology Center, Medistra Hospital, University of Indonesia, Jakarta, Indonesia; 18 Department of Internal Medicine, Cipto Mangunkusumo Hospital, University of Indonesia, Jakarta, Indonesia; 19 Third Department of Internal Medicine, Teikyo University School of Medicine, Chiba, Japan; 20 Department of Gastroenterology, Ankara University School of Medicine, Ankara, Turkey; 21 Department of Hepatology, Institute of Liver and Biliary Sciences, New Delhi, India

**Keywords:** Hepatocellular carcinoma, Asia–Pacific, APASL, Treatment algorithm

## Abstract

There is great geographical variation in the distribution of hepatocellular carcinoma (HCC), with the majority of all cases worldwide found in the Asia–Pacific region, where HCC is one of the leading public health problems. Since the “Toward Revision of the Asian Pacific Association for the Study of the Liver (APASL) HCC Guidelines” meeting held at the 25th annual conference of the APASL in Tokyo, the newest guidelines for the treatment of HCC published by the APASL has been discussed. This latest guidelines recommend evidence-based management of HCC and are considered suitable for universal use in the Asia–Pacific region, which has a diversity of medical environments.

## Introduction

Liver cancer is currently the second most common cause of cancer-related death worldwide [1], and hepatocellular carcinoma (HCC) accounts for more than 90% of liver cancers [2]. There has been a marked increase in HCC-related annual death rates during the past two decades, with the majority of all cases of HCC worldwide found in the Asia–Pacific region [3]. Thus, HCC represents a major public health problem in the Asia–Pacific region.

The Asian Pacific Association for the Study of the Liver (APASL) HCC guidelines were published in 2010 [4], being the oldest of the major guidelines. The “Toward Revision of the APASL HCC Guidelines” meeting was held at the 25th annual conference of the APASL in Tokyo on February 23, 2016. The attendees consisted of expert hepatologists, hepatobiliary surgeons, radiologists, and oncologists from the Asia–Pacific region. These members have discussed and debated the contents of the newest guideline. The new guideline is evidence based and is considered to be generally accepted in the Asia–Pacific region, which has a diversity of medical environments. The evidence and recommendations in the guideline have been graded according to the Grading of Recommendations Assessment, Development and Evaluation (GRADE) system (Table 1) [5, 6]. The finalized recommendations for the management of HCC are presented in this review.

**Table 1:** Grading of evidence and recommendations (adapted from the GRADE system [5, 6])

	Notes	Symbol
Grading of evidence		
High quality	Further research is very unlikely to change our confidence in the estimate of effect	A
Moderate quality	Further research is likely to have an important impact on our confidence in the estimate of effect and may change the estimate	B
Low or very low quality	Further research is very likely to have an important impact on our confidence in the estimate of effect and is likely to change the estimate. Any estimate of effect is uncertain	C
Grading of recommendation		
Strong recommendation warranted	Factors influencing the strength of the recommendation included the quality of the evidence, presumed important patient outcomes, and cost	1
Weaker recommendation	Variability in preferences and values, or more uncertainty: more likely a weak recommendation is warranted. Recommendation is made with less certainty; higher cost or resource consumption	2

## Epidemiology and risk factors

Liver cancer is the sixth most common cancer worldwide, being the fifth most common in males (7.5% of total) and the ninth in females (3.4% of total) [7]. Each year, approximately 78,200 new cases are diagnosed [7]. The prognosis for liver cancer is very poor (overall mortality to incidence rate, 0.95). The most frequent type is HCC, a cancer derived from liver hepatocytes. There are other types of liver cancer, such as intrahepatic cholangiocarcinoma (derived from biliary cells), sarcomas, and so forth, which should also be taken into account. Worldwide, approximately 80% of HCC cases are caused by hepatitis B virus (HBV) and/or hepatitis C virus (HCV) infection, especially in the setting of established cirrhosis or advanced fibrosis. The highest age-adjusted incidence rates (>20/100,000) are recorded in East Asia (North and South Korea, China, and Vietnam) and sub-Saharan Africa [8]. Approximately 75% of liver cancers occur in Asia, with China accounting for more than 50% of the world’s burden [9]. The incidence of HCC is likely to increase over the next 10–20 years and to peak around 2030.

The global age distribution of HCC varies by region, sex, and etiology. Globally, the rate of males suffering from HCC is higher than that of females, with male-to-female ratio ranging between 2:1 and 4:1, with the difference being much greater in high-risk areas. The sex disparity in rates is not well understood, although most liver cancer risk factors are more prevalent in males than females. Differences in sex steroid hormones, immune responses, and epigenetics could be related to the higher rates among males. These variations of age-specific patterns are likely related to the differences in the dominant hepatitis virus in the population, the age at viral infection, as well as the existence of other risk factors. In addition to sex differences, racial/ethnic disparity within multiethnic populations is also notable. Rates of liver cancer among persons of the same ethnicity also vary by geographic location; For example, liver cancer rates among Chinese populations outside China are lower than the rates reported by Chinese registries.

The single largest risk factor for development of HCC is cirrhosis of any etiology, which is present in 70–90% of those who have primary liver cancer [10]. In Africa and Asia, where HBV is endemic, 60% of HCC is associated with HBV, 20% is related to HCV, and the remaining is distributed among other risk factors. The risk of HCC developing among patients chronically infected with HBV ranges from 10- to 100-fold greater compared with the rates in uninfected people, depending on the markers and populations that are evaluated. In HCV infection, the relative risk (RR) for HCC developing in patients with serologically confirmed HCV infection is estimated to be 17-fold. The effect of high rates of alcohol abuse in Asia (as in the rest of the world) and the recent (10–15 year) obesity and type 2 diabetes mellitus (DM) epidemic in Asia may increase the HCC incidence in the next 25 years. In addition, patients who have multiple risk factors are not uncommon in the Asia–Pacific region [i.e., HBV/HCV, alcohol/HBV or HCV, DM/HBV or HCV, and HCV/human immunodeficiency virus (HIV)].

The incidence of HCC has remained the same over the last 20 years in most Asian–Pacific countries, except Singapore, where the incidence for males and females has fallen over the last 30 years. China and Taiwan have reported increasing incidence of HCC for males and females. This may be due to increasing awareness of reporting and better screening services. The country with the highest incidence rate, however, is Mongolia, with an age-standardized rate per 100,000 persons of 78.1.

Even within specific geographic regions, however, there is great variability. In Australia, as in the USA, traditionally very low-incidence regions, there has been a substantial increase (two- to threefold) in HCC incidence over the last 25 years, most probably due to immigration of people from the Asia–Pacific and other regions with high prevalence rates of chronic HBV infection, but also due to the epidemics of chronic HCV infection, possibly obesity, and DM.

## Risk factors for HCC

### HBV

Several meta-analyses have demonstrated that the risk of HCC is 15–20 times greater among HBV-infected individuals compared with the uninfected population [11]. Countries with chronic HBV infection prevalence greater than 2% have increased incidence and mortality rates of HCC [12]. Case–control studies in all regions of Asia have shown that chronic HBV infection is significantly more common among HCC cases than controls with odds ratios (ORs) ranging between 5:1 and 65:1 [13]. Similarly, prospective studies of HBV carriers have consistently demonstrated high RRs for HCC, ranging from 5 to 103 [14].

The lifetime risk of HCC among chronic HBV-infected patients is estimated to be 10–25% [15]. Several factors have been reported to increase the HCC risk among HBV carriers, including demographics (male sex, older age, Asian or African ancestry, family history of HCC), viral [higher levels of HBV replication; HBV genotype; longer duration of infection; coinfection with HCV, HIV, or hepatitis D virus (HDV)]; clinical (cirrhosis), and environmental or lifestyle factors (exposure to aflatoxin, heavy alcohol drinking, or tobacco smoking).

Risks of HCC among HBV-infected patients vary by several factors, the major one being serum HBV-DNA levels. Although there is no discrete cutoff level, having greater than Log_10_ 5/mL viral copies confers a 2.5- to threefold greater risk over an 8- to 10-year follow-up period than does having a lower viral load [14]. The cumulative incidence of HCC increases with serum HBV-DNA levels. A recent hospital-based cohort study further validated the HCC risk, showing it started to increase when the HBV-DNA level was higher than 2000 IU/mL [16].

In addition to HBV-DNA levels, the clinical significance of quantitative hepatitis B surface antigen (HBsAg) has become increasingly recognized. Data from the Risk Evaluation of Viral Load Elevation and Associated Liver Disease/Cancer–Hepatitis B Virus (REVEAL–HBV) and the ERADICATE-B study all showed that serum HBsAg ≥ 1000 IU/mL and HBV-DNA levels are complementary markers in predicting disease progression to cirrhosis and HCC [17, 18]. Cumulative HCC risk from age 30 to 70 years is estimated to be 87% for those persistently positive for HBsAg and hepatitis B envelope antigen (HBeAg), 12% for those persistently positive for HBsAg only, and 1% for those negative for HBsAg [19]. Therefore, prolonged duration of HBeAg positivity or high HBV-DNA levels may be associated with increased risk of HCC.

In multiple population-based studies, genotype C has been associated with higher risk of HCC than genotypes A, B, and D [20, 21]. In studies controlled for genotype, double mutations in the basal core promoter of the HBV genome were independent predictors of increased risk [22]. Mutations in the precore region of the viral genome also have been associated with risk, although less consistently so [23]. A study in Taiwan reported the importance of perinatal transmission of HBV and maternal virus load as a risk factor for HBV carcinogenesis in a familial clustering of HCC [24]. A family history of liver cancer, particularly among first-degree relatives, in HBV-infected individuals has been shown to increase the incidence of HCC.

Prevention of chronic HBV infection via vaccination drastically reduces the risk of HCC. In Taiwan, 30 years after the initiation of universal newborn vaccination, HBV carrier rates in persons younger than age 30 have decreased from 10–17% to 0.7–1.7% and rates of HCC have decreased by 80% [25].

Several host and viral factors predictive of HCC risk have been identified, and the Risk Estimation for Hepatocellular Carcinoma in Chronic Hepatitis B (REACH-B) study has developed and validated a predictive score for the risk of development of HCC in 3584 noncirrhotic chronic HBV Taiwanese and a validation cohort with 1050 patients with chronic HBV [26]. The 17-point risk score is composed of five predictors of HCC, including male sex, age, serum alanine aminotransferase (ALT) level, HBeAg status, and serum HBV-DNA level. The risk score can precisely estimate the risk of HCC development at 3, 5, and 10 years of follow-up.

Baseline liver stiffness values could be an independent predictor of HCC in patients with chronic HBV infection, where the 3-year cumulative incidence of HCC was significantly higher in patients with a higher liver stiffness value [27]. A recent Korean study included 1250 chronic HBV patients with baseline liver stiffness values to construct a predictive model for HCC occurrence based on a Cox proportional hazards model [28]. By using multivariate analysis, age, male sex, and liver stiffness values were independent predictors of HCC, whereas HBV-DNA levels >20,000 IU/L showed borderline statistical significance. A predictive model for HCC was developed using these four variables, with a correlation coefficient of 0.905 between predicted and observed risks of HCC occurrence.

It is currently clear that antiviral therapy reduces but does not eliminate the risk of HCC in chronic HBV patients with or without cirrhosis. Emerging data with the currently first-line nucleos(t)ide analogs, entecavir and tenofovir, suggest that the risk of HCC is also reduced under long-term therapies with these agents [29, 30]. The treatment benefit from the reduction of HCC incidence is always greater in patients with high baseline HCC risk, particularly those with cirrhosis. In addition, the reduction of HCC incidence under a high genetic barrier nucleos(t)ide analog is higher in the vast majority of patients who will achieve virological remission compared with those who may maintain detectable viral replication.

### HCV

Prospective studies have shown an increased risk of HCC in HCV-infected cohorts. In Japan, HCV antibody (HCV Ab)-positive cases of HCC accounted for more than 70% of cases diagnosed over the last 10 years [31]. Recently, its incidence has been decreasing. In Korea, approximately 10–20% of HCC patients are positive for HCV Ab. A meta-analysis of case–control studies showed that individuals positive for HCV Ab had 17 times the risk of HCC compared with the HCV Ab-negative cohort [32]. HCV appears to increase the risk of HCC by not only inducing hepatic inflammation and fibrosis, but also promoting malignant transformation of infected cells. The risk is highest among cases with cirrhosis where HCC develops at rate of 1–4% per year, though rates up to 8% have been reported in Japan [33]. The Hepatitis C Antiviral Long Term Treatment Against Cirrhosis (HALT-C) trial showed that 8% of patients without cirrhosis but with advanced fibrosis developed HCC [34]. Other risk factors that increase the risk of HCC in infected patients include male sex, coinfection with HIV or HBV, HCV genotype 1b, older age, presence of DM and obesity, and high level of chronic alcohol consumption. There is no consistent evidence that HCV viral load or quasispecies are important in determining the risk of progression to HCC.

### HBV/HCV coinfection

Three meta-analyses have confirmed that patients with dual HBV/HCV infection have an increased risk of HCC [11, 35, 36]. Different mechanisms have been hypothesized as being associated with development of HBV- or HCV-related HCC. Both viruses could play an active role at different steps of the carcinogenic process when they are present together in hepatocytes, and may be synergistic in causing HCC.

### Alcohol

A recent meta-analysis of 19 prospective studies estimated a 16% increased risk of liver cancer among consumers of three or more drinks per day and a 22% increased risk among consumers of six or more drinks per day [37]. Higher risks were found even for the lowest dose of alcohol (25 g/day), corresponding to approximately two drinks per day [38]. Chronic alcohol use of more than 80 g per day for longer than 10 years increases the risk for HCC by fivefold [32]. A recent meta-analysis showed a dose–response relationship between alcohol intake and liver cancer with RR of 1.19 [95% confidence interval (CI) 1.12–1.27], 1.40 (95% CI 1.25–1.56), and 1.81 (95% CI 1.50–2.19) for 25, 50, and 100 g of alcohol intake per day, respectively [39]. A study from the University of Michigan confirmed that alcohol consumption had a dose-dependent effect on the risk of HCC; the risk increased after 1500 g-years of alcohol exposure (60 g per day for at least 25 years) [40].

However, there is no safety limit for the effects of alcohol on the liver. In a study from Japan involving 804 HCC cases, the multivariate-adjusted hazard ratios (HRs; 95% CI) for alcohol intakes of 0.1–22.9, 23.0–45.9, 46.0–68.9, 69.0–91.9, and >92.0 g per day compared with occasional drinkers were 0.88 (0.57–1.36), 1.06 (0.70–1.62), 1.07 (0.69–1.66), 1.76 (1.08–2.87), and 1.66 (0.98–2.82), respectively [41]. In females who drank more than 23.0 g per day, a significantly increased risk was noted when compared with social drinkers (HR 3.60; 95% CI 1.22–10.66). A meta-analysis of four studies performed to assess the decline of liver cancer risk over time for former drinkers found that the risk of liver cancer falls after cessation by 6–7% a year, but an estimated time period of 23 years is required after drinking cessation before the risk returns to that of nondrinkers, with a large 95% CI of 14–70 years [42].

Alcohol acts synergistically with preexisting chronic liver disease, such as HCV, HBV, and fatty liver disease, as well as lifestyle choices, such as smoking and obesity, to further increase the risk of HCC in these disease states. In a retrospective cohort study by Berman et al. [43], the patients with cirrhosis due to a combination of HCV and alcohol had a significantly higher risk of HCC than those with cirrhosis due to alcohol alone (HR 11.2; 95% CI 2.3–55.0). Patients with HCV and alcohol exposure had a reduced tumor-free survival compared with those with HCV alone [44]. A multivariate analysis of 553 patients with HCC and 160 control subjects affected with HBV from China by Zhu et al. [45] revealed that heavy alcohol use, smoking, and positive family history of liver cancer are associated with HCC development among patients with HBV infection. A prospective case–control study of 210 subjects from the University of Michigan found that there was a dose-dependent relationship between alcohol and tobacco exposure with risk of HCC and synergistic index of 3.3 [40]. History of smoking and alcohol abuse worsened prognosis independently of each other, especially in viral hepatitis-related and early HCC. Abstinence from either reduced HCC-specific mortality, but only after 10 years of cessation [46]. An analysis of 2260 Taiwanese males from the REVEAL-HBV study cohort showed that the risk of HCC increased synergistically in alcohol users who had extreme obesity compared with those without extreme obesity and with nonusers of alcohol [47]. A study from Italy enrolled 465 HCC patients and compared them with 618 cirrhotic patients without HCC and 490 healthy controls, evaluating the association among DM and alcohol abuse in the HCC group versus both control groups. This study showed that, for alcohol abuse alone, the OR for HCC was 3.7 (95% CI 2.5–5.4) and 49.0 (95% CI 21.5–111.8) in DM with significant alcohol intake [48].

### Nonalcoholic fatty liver disease (NAFLD)

Meta-analyses of DM and HCC have consistently estimated RRs of 2.0–2.5 and have found that the relationship is consistent across various populations and is independent of other risk factors [49–51]. Several studies have reported that obesity is also related to liver cancer [52]. In comparing normal-weight persons with overweight and obese persons, a meta-analysis of 11 cohort studies found significant liver cancer risks among overweight and obese persons [53]. Similarly, a meta-analysis of four studies of metabolic syndrome and HCC estimated a significant RR of 1.81 [54]. Although the RRs of DM, obesity, and metabolic syndrome do not approach those of HCV or HBV, they are far more prevalent conditions than HCV and HBV in developed countries. Given the increasing prevalence of these conditions, the proportion of HCC related to obesity, DM, and metabolic syndrome will likely increase in the future.

The results of recent studies demonstrated that HCC is more prevalent in the setting of obesity and insulin resistance, and may occur in nonalcoholic fatty liver disease (NAFLD) patients without cirrhosis. Indeed, in a recent retrospective cohort study that evaluated trends in HCC etiology among adult recipients of liver transplantation (LT) from 2002 to 2012, the number of patients undergoing LT for HCC secondary to nonalcoholic steatohepatitis (NASH) increased by nearly fourfold, whereas the number of patients with HCC secondary to HCV increased by only twofold [55]. Available data suggest that obesity increases the risk of HCC 1.5- to fourfold. One large meta-analysis included seven cohort studies of 5037 overweight subjects (body mass index 25–30 kg/m^2^) and ten studies of 6042 obese subjects (body mass index >30 kg/m^2^); compared with normal-weight people, HCC risk increased 17% in those who were overweight and 89% in those who were obese [53]. In a study from Japan that looked at the recurrence of HCC after ablation therapy in NASH patients, increased visceral fat was an independent risk factor for recurrence of HCC at 3 years (75.1 versus 43.1% with low visceral fat) [56]. In a similar trend seen in many studies, type 2 DM was associated with a substantially increased risk of HCC. Although it is possible that the increased HCC risk associated with DM seen in these studies may be mediated through the development of NAFLD, the presence of multiple pathogenic mechanisms common to obesity, insulin resistance, and NAFLD suggests that this link may not be mediated through NAFLD per se.

Evidence of the development of HCC in noncirrhotic patients continues to accumulate in case reports or case series. Obesity, insulin resistance, and the proinflammatory milieu of NASH may mediate carcinogenesis directly. In a recent study analyzing 1419 HCC cases that were related to NASH (120 cases), HCV (1013 cases), and alcohol (286 cases), cirrhosis was present in only 58.3% of NASH-related HCC cases [57]. Limited available data suggest that risk factors for the development of NASH without cirrhosis include older age, male sex, and metabolic syndrome [58]. In a study of 87 Japanese NASH patients with HCC, Yasui et al. [59] found that 56% of cases were noncirrhotic, and it was noted that males developed HCC at a less advanced stage of liver fibrosis than females. Hashimoto et al. [58] examined 34 cases of NASH-related HCC and found that there was a prevalence of advanced age, male sex, obesity, and type 2 DM; 12% of the patients had stage 1 or 2 fibrosis, and 88% had advanced fibrosis (stage 3–4). These HCC patients tended to be older, male, and have metabolic syndrome.

### Budd–Chiari syndrome

Twelve studies were conducted in Asian countries between 1958 and 2008 to evaluate the prevalence of HCC in Budd–Chiari syndrome patients; the pooled prevalence of HCC was 17.6% in Budd–Chiari syndrome patients and 26.5% in inferior vena cava obstruction patients [60]. There was no statistically significant difference in sex, age, or site of obstruction between HCC and non-HCC groups. It has not been clarified whether HCC occurred only in cirrhosis patients. The prevalence of HCC in Budd–Chiari syndrome patients is highly variable, ranging from 2.0 to 51.6%, as the diagnostic criteria and the methods are significantly discrepant among studies, which potentially influence the prevalence of HCC, and the length of follow-up was also different [61, 62]. In addition, it is necessary to consider carefully that occurrence of HCC is difficult to identify using dynamic computed tomography (CT) or dynamic magnetic resonance imaging (MRI) because of the changes in venous drainage associated with venous outflow obstruction. Further studies are necessary to evaluate the risk factors for HCC in Budd–Chiari syndrome.

### HCC in other liver diseases

Other notable causes of cirrhosis can increase the risk of the development of HCC. In patients with genetic hemochromatosis, in whom cirrhosis is established, the RR for liver cancer is approximately 20-fold higher. The incidence of liver cancer in individuals with stage 4 primary biliary cholangitis (PBC) is similar to the incidence in patients with HCV and cirrhosis, and this suggests that PBC confers a high risk for HCC [63]. Patients with autoimmune hepatitis and cirrhosis also have an increased incidence of liver cancer [64]. In addition, HCC has been reported in patients with Wilson disease. However, a recent report indicated that the risk of HCC was low in Wilson disease even in cases of cirrhosis [65].

### Host genetics

HCC develops in only a small percentage of those infected with HCV or HBV. Host genetic makeup may be an important factor that influences progression to HCC. Two meta-analyses identified variants of tumor necrosis factor (TNF) associated with a higher risk of HCC [66, 67]. They showed that TNFα-308 AA and AG variants (versus GG) were associated with a significantly increased risk of HCC. A recent meta-analysis concluded that null genotypes of glutathione *S*-transferase (GST) genes (*GSTM1* or *GSTT1*) were associated with an increased risk of HCC [68].

### Aflatoxin

Aflatoxin B1 is a major hepatocarcinogen [69], which acts in part by causing mutations of codon 249, a mutational hotspot of the p53 tumor suppressor gene. Aflatoxin B1 exposure, however, is more common in areas where HBV is the dominant virus, including sub-Saharan Africa, Southeast Asia, and China. Within these areas, higher levels are found among rural than urban populations [70], among males than females [71], and among persons chronically infected with HBV [72]. Aflatoxin B1 is metabolized by CYP2E1, which is induced by alcohol. Thus, alcohol may have an incremental genotoxic effect on aflatoxin B1. One case–control study suggests that combining aflatoxin B1 load and alcohol intake has a synergistic and a statistically significant effect on RR (RR = 35) [73]. There is a synergistic association between aflatoxin B1 and HBV in increasing the risk of HCC. Compared with persons with neither risk factor, the risk of HCC is reported to be fourfold greater among persons with elevated levels of aflatoxin B1, sevenfold greater among chronic HBV carriers, and 60-fold greater among persons with both factors [74, 75]. Evidence suggests that there is also a synergistic effect between aflatoxin B1 and HCV infection [76].

### Tobacco

In 2004, the International Agency for Research on Cancer (IARC) concluded that there was sufficient evidence that tobacco smoking increased the risk of liver cancer [77]. A recent meta-analysis estimated that there was a 1.5-fold increased risk of HCC among current smokers, a risk similar to that imposed by obesity [78]. Inconsistent findings in studies of the same populations, and the correlation of smoking with other risk factors, such as HBV, HCV infection, and alcohol consumption, have made the relationship between tobacco and HCC difficult to define.

### Coffee and tea

Recent meta-analyses have examined the association between coffee and tea and the risk of HCC [79, 80]. The coffee meta-analysis found a significant 40% reduced risk of HCC among consumers, and tea was associated with a nonsignificant 23% reduced risk. Compounds in coffee that potentially have chemopreventive effects include diterpenes, chlorogenic acid, and caffeine [81]. Diterpenes are lipids that inhibit enzyme expression and enzymatic activity, induce detoxifying enzymes, and regulate signaling pathways [82]. Chlorogenic acid is a polyphenol that increases the activity of detoxifying enzymes [83]. Caffeine has antioxidant properties and increases the metabolic rate and energy expenditure, which could potentially regulate weight and reduce the risk of developing metabolic syndrome [84]. Similarly, tea contains bioactive compounds, including caffeine and polyphenolic compounds. One specific polyphenol, epigallocatechin-3-gallate, has shown promise as a chemopreventive agent by inhibiting enzymatic activities, cell invasion, angiogenesis, and metastasis [85].

### HCC in children

HCC is rare among adolescents and accounts for less than 1% of all malignant neoplasms among children younger than 20 years [86]. Hence, the risk factors are not well studied. Hepatoblastoma is the most common primary hepatic malignancy (48%); HCC is the second most common primary liver malignancy of childhood (27%), with vascular tumors and sarcomas making up the rest. HCC has an incidence of 0.3–0.45 cases per million per year and represents an increasingly common indication for LT in children [87]. HCC is more common in adolescents (10–14 years), more common in males than in females with a 3:1 preponderance, and tends to present with more advanced disease in children than in adults. HCC incidence increases significantly with age. Overall, only 0.5–1% of cases occur before the age of 20 years. The incidence of HCC in chronic HBV carriers is approximately 100-fold greater than that in the HBV-negative population and is more common in areas with high endemic HBV infection rates [88].

The decrease of HBV because of neonatal vaccination has led to a reduction of cases in childhood, which will, in time, be reflected in the adult population [89]. Although HCV is a known risk factor for HCC in adults, it is rare in children, and there is only a single case report of this occurrence requiring a transplant [90].

Inherited metabolic disorders, specifically hereditary tyrosinemia, α-1-antitrypsin deficiency, and glycogen storage disease type 1, are associated with childhood cirrhosis and HCC. Tyrosinemia I (fumarylacetoacetate hydrolase deficiency) is an autosomal recessive inborn error of tyrosine metabolism that leads to liver failure in infancy or chronic liver disease with cirrhosis. Without treatment, there is a high risk of HCC in childhood or early adolescence. HCC is also associated with glycogen storage disease types I and IV [91].

Only approximately 30% of pediatric cases of HCC are associated with cirrhosis or preexisting liver abnormality, in contrast to adult HCC in which cirrhosis is present in 70–90% of cases. Similarly, α-1-antitrypsin deficiency exhibits a different mechanism for carcinogenesis, where liver injury results from abnormal and chronic regenerative signaling from the sick cells to younger, less sick hepatocytes: chronic regeneration in the presence of tissue injury leading to adenomas and ultimately to carcinomas. It was recently suggested that progressive familial intrahepatic cholestasis type 2 (PFIC 2), associated with a mutation of the *ABCB11* gene resulting in deficiency of bile salt export pump (BSEP; a membrane canalicular bile acid transporter), represents a specific and previously unrecognized risk for HCC in young children [92].

## Epidemiology of HCC in Asia–Pacific countries

### Japan

In Japan, HCC ranks as the fifth most common cancer, being the fourth most common in males and the sixth in females. Nationwide follow-up surveys by the Liver Cancer Study Group of Japan (LCSGJ) show that the age-standardized incidence rate of HCC and total number of deaths from HCC in Japan in males have shown a gradual declining trend since 2004 [7]. In 2012, a total of 30,690 people died of liver cancer in Japan. Although Japan is one of the Asia–Pacific countries with a high HCC incidence rate, the cause of HCC in Japan differs greatly from other countries in the region. Chronic HCV infection is more common than chronic HBV infection in Japan; chronic HCV infection accounts for 64.7% of HCCs. Chronic HBV infection, on the other hand, accounts for only 15.1% of HCCs [93]. In the near future, the prevalence of HCV-related HCC is expected to decrease because of the falling prevalence of HCV and deaths of older HCV patients from unrelated causes [94].

Japan has created the world’s first nationwide HCC surveillance program. Japan introduced a liver cancer screening program as early as the 1980s. In 1999, the Japan Society of Hepatology (JSH) began the Eliminate Liver Cancer Program [95]. In addition, the Basic Act on Hepatitis Measures enacted by the Japanese Ministry of Health, Labour and Welfare in 2009 established a system by which public health centers and clinics could perform blood tests free of charge for the general public to check for infection with HBV or HCV. Other possible reasons for the declining incidence rate may be the great success of postnatal HBV vaccination, screening of donated blood, and efforts to educate the general public about HCV.

### India

In India, information on HCC is inadequate. Based on cancer registries in five Indian urban populations (Mumbai, Bangalore, Chennai, Delhi, and Bhopal) over a period of two decades, liver cancer ranks as the fifth most frequent cancer for both sexes [96]. However, the cancer registries in India probably do not provide accurate estimates of HCC prevalence due to their predominantly urban location and because the sources of information on cancers are from cytology, oncology sites, and municipal registers of death. The available data indicate that the age-adjusted incidence rate of HCC in India for males ranges from 0.7 to 7.5 and for females from 0.2 to 2.2 per 100,000 population per year [97]. The incidence of HCC in patients with cirrhosis in India is 1.6% per year. The male-to-female ratio for HCC in India is 4:1. The age of presentation varies from 40 to 70 years. The age-standardized mortality rate for HCC in India for males is 6.8 per 100,000 population and for females is 5.1 per 100,000 population. In India, HBV and HCV infection and alcohol consumption are the main causes of HCC [98]. Reports from tertiary care centers in India on HCC indicate that 70–97% of patients with HCC had underlying cirrhosis of the liver at the time of diagnosis. Approximately one-quarter of HCC cases diagnosed in India do not have any known predisposing risk factors [99]. The presence of any HBV marker (HBsAg positive or presence of HBV antibodies even in absence of HBsAg) increases the risk of HCC [100]. Moreover, huge regional differences in the prevalence of HBV and HCV infection might exist in India (i.e., the prevalence of HCV infection was the highest in the Punjab area). These differences might translate into large differences in the incidence of HCC between states. Because of the discrepant and isolated reports on genetic risk factors for HCC, the data are currently insufficient to implicate any genetic risk factor for HCC in India. The unpublished data from various tertiary care centers suggest that the incidence of HCC is increasing in India.

### Australia

Liver cancer is relatively uncommon in Australia, where it ranks 15th in males and 20th in females [101]. However, over the last three decades, HCC incidence rates have been rising in Australia, both from cases attributed to HCV and from HBV, the latter related to migration from high-prevalence countries [101]. Data from the New South Wales Cancer Registry indicate that age-standardized primary liver cancer incidence rates have increased from 2.0 and 0.5 per 100,000 population in 1972 in males and females, respectively, to 7.4 and 2.9 per 100,000 population in 2004 [102]. Other known risk factors for the increasing incidence of HCC include HBV/HCV coinfection and cirrhosis due to various other causes. According to an Australian study of HCC incidence as stratified by different chronic liver diseases, the HCC incidence rate of patients with HBV monoinfection was markedly higher than that of those with HCV monoinfection (9.5 versus 6.9 cases per 10,000 population-years) [103]. A recent population-based linkage study showed that Asian-born residents with chronic HBV were 30 times more likely to suffer HCC compared with Australian-born residents [104]. The incidence of HCC with chronic viral hepatitis is associated with increasing age, male sex, and other comorbidities. The highest age-specific risk of developing HCC occurs among people aged 75 years and older, being over 14 times the risk for those aged under 45 years. The risk in male patients is threefold higher than that in females [103].

### China

Liver cancer is the second most common cancer in China. Overall, the estimated incidence rate of HCC is 40.0 in males and 15.3 in females per 100,000 population. HCC is ranked as the second most common cause of cancer mortality in males after lung cancer, while it ranked third in females, after lung and gastric cancer. Approximately 383,203 persons die of liver cancer every year in China, which accounts for 51% of deaths from liver cancer worldwide. The mortality rate of liver cancer is higher in males (37.4/100,000) than in females (14.3/100,000) [105]. In China, some identified risk factors, such as HBV, HCV, aflatoxin B1, alcohol consumption, and tobacco smoking, contribute to the incidence and mortality related to HCC. In particular, HBV infection contributes to large number of liver cancer deaths and cases (63.9%). For HCV, its rate in liver cancer deaths and cases is lower than that of HBV (27.7% overall; 27.3% in males and 28.6% in females), but is still higher than that of aflatoxin exposure (25% of the population), alcohol drinking (15.7%), and tobacco smoking (13.9%) [106].

Despite the high incidence of liver cancer throughout China, a decreasing trend has been observed in some regions because of neonatal vaccination for HBV infection. According to the IARC and Cancer Incidence in Five Continents, the age-adjusted incidence rate of HCC has been in on decline in Shanghai since the 1970s and in Tianjin since the 1980s [107, 108].

### Hong Kong

According to the report from the Hong Kong Cancer Registry 2012, liver cancer ranks as the fourth most common cancer and the third most common cause of cancer death. The incidence and mortality of liver cancer are higher in males (fourth and third, respectively, among all cancers) than in females (tenth and fourth, respectively, among all cancers). In Hong Kong, the incidence of HCC increases with age, and the highest age-specific rate occurs among people aged 75 years and older, accounting for 152.4 per 100,000 population. However, over the past 25 years, the incidence of HCC in different age groups (especially age >40 years) has shown an apparently downward trend, which may be explained partly by the declining rate of HBV infection due to the institution of universal HBV vaccination since 1988. Since 1992, chronic HBV has been the major cause of HCC in Hong Kong, accounting for 80% cases in 1992 and 78% cases in 2006. From 1992 to 2006, the proportion of HCV-related HCC increased from 3 to 6.3% [109].

### Korea

In South Korea, liver cancer is the fourth most common cancer in males and the sixth most common cancer in females. The age-standardized incidence rate is 46.5 per 100,000 population (males, 45/100,000; females, 12/100,000). The incidence increases for age over 40 years, reaching a peak at age of 55 years [110]. However, the incidence of liver cancer among Korean males and females declined from 1999 to 2010. HBV is the most common infectious etiologic factor for liver cancer in Korea (70–80%), followed by HCV. HCC is the third leading cause of cancer mortality in Korea. The successful changes in the rates of liver cancer mortality in Korea were not solely due to Korea’s HBV vaccination efforts, but also depended on its 10-year plan for cancer control, implemented by the government in 1996. With the introduction of the National Cancer Screening Program (NCSP) in 1999, males and females aged over 40 years with chronic hepatitis (HBsAg or HCV Ab positive) and liver cirrhosis patients, regardless of HBV or HCV infection, are offered screening for HCC.

### New Zealand

New Zealand cancer census data from 1981 to 2004 indicated that the age-standardized incidence rate of HCC was 30.3 per 100,000 population in Pacific Islander males and 9.8 per 100,000 population in Pacific Islander females, compared with 4.1 per 100,000 population and 2.1 per 100,000 population for their European counterparts. This suggests that the rate of HCC in the Pacific is 7 and 4 times higher than that in Europe for males and females, respectively [111].

### Taiwan

A survey from the Taiwan Cancer Registration System documented that the incidence rate of HCC had increased gradually in 1994 to 2007. The rate in males was higher than that in females. HBV infection is the most important cause of HCC, but this phenomenon is changing [112]. From 1981 to 1985, HBV-related HCC accounted for 88% of cases, whereas from 1995 to 2000, the proportion of HBV-related HCC had decreased to 59%, whereas the proportion of HCV-related HCC had increased to 31%. For HBV-related HCC, the ratio between males and females was 6.4, whereas for HCV-related HCC, it was 1.7 [113].

### Iran

Although the true prevalence of HCC in Iran is unknown, it is considered to be a low-risk area for HCC with an incidence less than five per 100,000 population. In contrast to Western countries, alcohol consumption has a minor role in HCC development in Iran. A study on the risk factors of HCC in southern Iran revealed that only 2.8% of HCC patients had history of excess alcohol intake. The same study showed that the predominant cause of HCC in the studied group was HBV followed by HCV infections with incidence of 52.1 and 8.5%, respectively. Approximately 80% of HCC patients were positive for at least one of the known HBV markers. Thus, HBV infection appears to be the most common cause of HCC in Iran [114].

### Pakistan

Unfortunately, no population-based study was available from which a true prevalence and incidence rate of HCC could be ascertained. Most of the studies were hospital based, consisting of case series with small sample size, or they had a highly selected population. However, a few cancer registries have been established in Pakistan. From the 1970s until the mid-1990s, HBV was the most common etiologic factor for HCC in Pakistan. Afterwards, a shift in HCC etiology was observed with a steady rise in HCV-related HCC cases. The age-standardized rate for HCC is 7.64 per 100,000 population in males and 2.8 per 100,000 population in females. The male-to-female ratio is 3.6:1. The usual age of presentation is in the fifth and sixth decades [115].

### Vietnam

Liver cancer is the leading cause of cancer-related death in males and the second most common for females in Vietnam [116]. Vietnam is a country with a high prevalence of HBV infection (an estimated 12.3% of males and 8.8% of females are chronically infected with HBV). Thus, HBV was the most common etiologic factor for HCC in Vietnam [117]. A recent study reported that the estimated chronic HBV prevalence increased from 6.4 million cases in 1990 to approximately 8.4 million cases in 2005 and was projected to decrease to 8.0 million by 2025 [118]. However, the estimated HBV-related HCC incidence increased from 9400 in 1990 to 25,000 in 2025. Although universal infant HBV vaccination will reduce the chronic HBV prevalence in Vietnam over the next two decades, the HBV-related HCC burden will continue to rise.

### Mongolia

The estimated incidence rate of HCC in Mongolia is 54.1 per 100,000 world standard population, one of the highest worldwide [119]. Although universal vaccination for HBV has been implemented and sterilization of medical devices is being improved, the prevalence of chronic HBV and HCV infection is still over 10%. HCV-related HCCs are more common than HBV-related cancers. In addition, coinfection with HBV and HCV occurs frequently [117]. Due to the lack of a surveillance system, the majority of cancers are diagnosed in advanced stages.

### Other countries

Liver cancer is one of the most common causes of cancer-related death in other Southeast Asian countries, such as Cambodia, Lao People’s Democratic Republic, Myanmar, and Papua New Guinea [116]. Although there is little epidemiologic information from those countries in regard to HCC, it is assumed that the high prevalence rate of HBV infection is related to the occurrence of HCC.

## Summary

Although it is difficult to accurately predict future changes in disease epidemiology, the overall global incidence of HCC is predicted to rise in the next few years until a plateau is reached by 2020. Subsequent decreases in the rates of HCC have been predicted, resulting at least in part from expected improvements in the control of HBV and HCV infection. However, as the contributions of HBV and HCV diminish, other risk factors, such as DM and obesity, may become increasingly important drivers of future HCC incidence trends.

## Prevention

### Prevention of HBV-related HCC

#### Recommendations

1. As primary prophylaxis for HCC, universal HBV vaccination in infants should be implemented in all countries, especially in HBV-endemic areas (A1).
2. As secondary prophylaxis for HCC development, effective and potentially long-term antiviral therapy should be started in all patients with chronic hepatitis B infection and active liver disease (B1).

The most common risk factor for HCC is chronic HBV infection, which accounts for more than 50% of all cases globally and 60–80% in some Asian countries such as China, Korea, and Vietnam [120]. Strategies to prevent HBV-related HCC include universal hepatitis B vaccination to reduce new infection as primary prevention, antiviral treatment to prevent disease progression by effectively suppressing HBV replication and regular surveillance to detect HCC in earlier stage as secondary prevention, and combination of curative therapies and adjuvant antiviral treatment to increase survival and prevent recurrence for HCC patients as tertiary prevention [2, 121, 122].

### Primary prophylaxis of HBV-related HCC: vaccination to decrease the rate of HBV infection

The aim of primary prophylaxis in HBV-related HCC is to prevent new HBV infection in healthy individuals. The universal vaccination programs carried out in countries with endemic HBV have resulted in a significant decline in the prevalence rate of HBsAg and incidence of HCC [10]. As an excellent example, the universal hepatitis B immunization program for newborns started in 1984 in Taiwan has significantly reduced the prevalence rates of HBsAg, acute and chronic hepatitis B, and cirrhosis, decreasing HCC incidence by more than 80% and more than 90% among cohorts vaccinated at birth, over 30 years [123, 124]. Similarly, a national survey showed that the prevalence of HBsAg declined from 9.75% in 1992 to 7.18% in 2006 and among children younger than 5 years old declined from 9.67% in 1992 to 0.96% in 2006 in Mainland China, where universal infant HBV vaccination started in 1992 [125]. More importantly, a recent report of a 30-year follow-up study demonstrates that the HCC incidence rate also decreased by 84% in vaccinated cohort in Qidong area of eastern China [126].

### Secondary prophylaxis of HBV-related HCC: antiviral treatment to reduce incidence of HCC in chronic HBV infection

Studies have revealed that a variety of factors are involved in HCC occurrence among chronic hepatitis B patients, including demographic, viral, and environmental factors. A large-scale cohort study (REVEAL-HBV study) carried out in Taiwan demonstrated that the incidence rate of HCC was correlated with serum viral load (1.3 and 14.9% for HBV-DNA < 300 copies/mL and ≥1,000,000 copies/mL, respectively) during a mean follow-up of 11.4 years [127]. Even patients with moderate HBV-DNA level (60–2000 IU/mL) also had a substantially increased risk of HCC and mortality compared with uninfected individuals [128].

Many studies have shown that antiviral treatment can decrease the incidence of HCC. A landmark randomized control trial (RCT), the Cirrhosis and Lamivudine Monotherapy (CALM) study, showed that, compared with placebo group, lamivudine therapy significantly reduced the risk of HCC in chronic hepatitis B patients with advanced fibrosis and cirrhosis (7.4 versus 3.9%) [129]. Meta-analyses confirmed the beneficial effect of antiviral treatment on reducing HCC risk, no matter whether using lamivudine, adefovir, entecavir, tenofovir or interferon [130, 131]. However, virological response was related to the clinical outcome of patients. The incidence of HCC in patients with sustained viral suppression was significantly lower compared with patients with suboptimal response [132]. A follow-up study showed that entecavir is more effective than lamivudine in prevention of HCC due to higher potency and minimal risk of resistance, with 5-year cumulative incidence of HCC of 7 and 20%, respectively [133]. Furthermore, the preventive efficacy of antiviral therapy can be translated to general population; for example, since launched in 2003, a viral hepatitis therapy program has significantly reduced incidence and mortality of HCC in the general population of Taiwan (HR was 0.86 for HCC incidence and 0.76 for HCC mortality) [134]. Of note is that suppression of viral replication in chronic hepatitis B patients by antiviral treatment could reduce but not eliminate the risk of HCC, especially in patients with cirrhosis [135–137]. Kim et al. reported that the risk of HCC remained after HBsAg seroclearance in chronic hepatitis B patients, especially in males, those who achieved HBsAg seroclearance at >50 years, and those who had liver cirrhosis [138]. Therefore, regular surveillance is important in patients receiving antiviral therapy, even in patients who lost HBsAg due to tumor detection at early stage.

### Prevention of HCV-related HCC

#### Recommendations

1. In chronic HCV infection, patients who obtained sustained virologic response (SVR) had considerably reduced risk of HCC. However, older age, low platelet count, and/or presence of cirrhosis despite SVR are associated with higher risk for HCC development and warrant surveillance (A1).

In chronic HCV infection, a meta-analysis of retrospective studies implies that the risk of HCC is reduced among patients with HCV who achieve SVR with antiviral therapy with interferon or interferon plus ribavirin [139–142]. Findings on the effect of SVR on liver-related clinical outcomes are similar to those of retrospective, and often smaller, studies from Japan [143–147], the results of which supported an approximately 70–90% reduction in the risk of liver-related clinical outcomes over a follow-up period of 2–6 years in patients achieving SVR. This was reaffirmed by a recent study on 33,005 HCV-infected individuals who received treatment, whose authors concluded that the risk of HCC after HCV cure, though considerably reduced, remains relatively high at 0.33% per year [148].

According to the HALT-C and EPIC studies, a continued elevated risk of HCC in patients with advanced chronic HCV, even in those who achieved SVR, was evident [139]. The 5-year risk of HCC developing in noncirrhotic patients was 4.8%. It has been suggested that the incidence of HCC in patients with cirrhosis from HCV only increases substantially once the platelet count is less than 100 × 10^9^/L. Furthermore, older age and presence of cirrhosis at the point of SVR are associated with a higher risk of HCC, warranting surveillance [148].

Although these studies have validated that there is a reduced incidence of HCC in treated patients, there are no data that demonstrate that treating or eradicating HCV completely eliminates the risk for HCC. Thus, it seems prudent to continue surveillance of patients with HCV and cirrhosis who have achieved viral clearance on therapy. Surveillance is recommended in SVR patients with any histologic stage of HCV with comorbidities, such as alcohol abuse and DM, all of which are established independent risk factors for HCC disease progression [148–151].

While the most commonly observed clinical benefits in SVR patients were the consequence of the arrest of fibrosis progression, regression of preexisting cirrhosis could be documented [152–157]. However, regressed cirrhosis is not a reason to withhold surveillance. The recent new arrival of direct-acting antiviral (DAA) therapy allowed for the achievement of SVR in over 90% of treated patients, irrespective of liver fibrosis stage [158–160]. However, there is no evidence that successful DAA therapy reduces the incidence of HCC in patients with HCV cirrhosis. Maintaining surveillance for SVR patients with advanced liver fibrosis, independently of the histologic response to therapy, is highly recommended.

### Prevention of metabolic-related HCC

#### Recommendations

1. Nonalcoholic fatty liver disease (NAFLD) and nonalcoholic steatohepatitis (NASH) are associated with a significant risk of HCC development, which is higher in the presence of cirrhosis (A2).
2. A significant proportion of patients may suffer HCC even in the absence of cirrhosis (B2).
3. Metabolic syndrome and its components, especially DM and obesity, are associated with a high risk of HCC in patients with NASH (A2).

The estimated prevalence of NASH in the general population ranges from 2 to 3% [161]. Contrary to earlier studies, recent data revealed that up to 44% of cases of NAFLD can progress to NASH even in the absence of inflammation at baseline [162] and approximately 23% of cases of NASH progress to cirrhosis over the next 10–15 years [163]. In general, up to 30–40% and 10–15% of cases of NASH do have advanced fibrosis and cirrhosis, respectively, at initial diagnosis [163]. Although the overall incidence of HCC depends on the stage of underlying NAFLD and associated comorbid conditions, HCC can develop in the absence of cirrhosis in such cases [164]. The incidence of HCC in patients with NASH cirrhosis has been reported to be 2.3–4.0% per year [165, 166].

Metabolic syndrome and components of metabolic syndrome have been associated with the development of HCC [167]. In a meta-analysis of 38,940 cases of cancers (43 articles), Esposito et al. [168] found an association of metabolic syndrome with HCC (RR = 1.43, *p* < 0.0001), and the association was stronger in Asians (*p* = 0.002). Moreover, the estimated risk of HCC was high in overweight (RR = 1.48; 95% CI 1.31–1.67) and obese (RR = 1.83; 95% CI 1.59–2.11) individuals. Tanaka et al. [169] reported that overweight or obesity increased the risk of liver cancer among Japanese population in the meta-analysis. Consistent with these findings, a significant association of DM and HCC was estimated (RR = 2.31; 95% CI 1.87–2.84) by Wang et al. in a meta-analysis of 17 case–control and 32 cohort studies [170]. Longer duration of DM and treatment with insulin or sulfonylureas was also associated with a higher risk of HCC, while a lower risk of HCC was found with metformin treatment. In another meta-analysis of 25 cohort studies, a higher incidence of HCC was found in 17 studies [summary relative risks (SRRs) = 2.01; 95% CI 1.61–2.51] among diabetics compared with nondiabetic patients. However, due to the presence of significant heterogeneity among studies (*Q* = 136.68, *p* < 0.001, *I* ^2^ = 87.6%), a subgroup analysis was performed to control for confounders and DM was associated with a higher HCC-related mortality (SRR = 1.56; 95% CI 1.30–1.87) [51].

Because HBV and HCV are predominant risk factors responsible for a high burden of liver diseases in the Asia–Pacific region, Chen et al. [171] evaluated the influence of obesity, DM and HBV/HCV infections on the risk of HCC. This meta-analysis found that the positive association with obesity was independent of DM or infections with HBV/HCV.

In general, the relationship between DM, obesity, metabolic syndrome, and HCC is linked with development of NAFLD, and theoretically all are interlinked with each other. Considering the significant burden of NASH (i.e., 2–3% of the global population) and a global rise in the burden of obesity and DM, it is expected that there will be a further increase in the burden of NASH and HCC in the foreseeable future unless considerable preventive measures are taken [172]. Most of the studies have used the Western criteria for obesity, and one can expect an even higher risk if similar estimates are calculated using the Asian criteria for obesity. Higher HCC-related mortality rates have been reported in DM compared with non-DM patients (RR = 2.43; 95% CI 1.66–3.55) [170]. Furthermore, due to limited available treatment options, patients with NASH-related cirrhosis carry a significant risk for HCC development. Moreover, many other aspects must be explored; for instance, the role of impaired glucose metabolism, dyslipidemia, and the effect of concomitant use of alcohol must be evaluated [167]. Hence, it is imperative to implement measures to reduce the burden of factors associated with NAFLD/NASH.

So far, the major key preventive measures here include “healthier diet” and lifestyle modification, which should be explained and promoted to every individual who is at risk of or has suffered from such metabolic derangements. Dietary modifications according to underlying risk factors, such as DM, obesity, dyslipidemia, and hypertension, should be promoted. Regular walking and exercise also have a major role in the control of metabolic syndrome and NAFLD/NASH. Treatment of concomitant metabolic conditions with statins and metformin may also have beneficial effects on portal hypertension, complications of liver cirrhosis, and HCC prevention [173]. The efficacy of metformin as a preventive agent in a clinically relevant rat model of HCC was evaluated and was associated with a reduction in fibrotic and inflammatory markers and a 44% decrease in HCC incidence when administered in an early phase by suppressing the receptors for advanced glycation end products and inhibiting activation of hepatic progenitor cells [174]. However, these preclinical findings must be confirmed in clinical studies.

Bariatric surgery could be recommended for patients with morbid obesity, which may reduce liver fibrosis but carries a risk of decompensation in patients with advanced liver cirrhosis [173]. Furthermore, periodic screening for HCC in patients with NASH will help to identify HCC cases at early stage. Patients with NASH cirrhosis should be considered for HCC screening according to the American Association for the Study of Liver Diseases (AASLD)/American College of Gastroenterology (ACG) practice guidelines [175]. The other systemic review of NAFLD recommended biannual imaging screening in cirrhosis patients [176]. However, screening recommendations have not yet accounted for the increasing number of patients suffering from HCC even though up to 50% of cases may occur in the absence of cirrhosis [59]. The latest European guidelines suggested that the PNPLA3 rs738409 *C* > *G* gene polymorphism has been associated with an increased HCC risk and might provide patient risk stratification for tailored HCC surveillance in NAFLD. However, no recommendation can currently be made on the timing of surveillance or its cost-effectiveness [177].

### Tertiary prevention

#### Recommendations

1. Interferon did not reduce the recurrence-free survival (RFS) rate in HBV-related HCC after curative treatment. However, it is possible it improved overall survival (OS) (A2).
2. Nucleos(t)ide analogs may be effective in reducing the risk of recurrent HBV-related HCC after curative treatment (B2).
3. Interferon-based antiviral treatments after curative therapy in HCV-related HCC may reduce the risk of recurrence and improve survival rates (A2).

### Tertiary prevention for HBV-related HCC

HBV viral load has been shown to have an important role in carcinogenesis in patients with chronic HBV liver disease, and recently, HBV viral load has also been reported to be involved in recurrence after radical treatment of HCC [178]. In a retrospective study of 72 patients and a prospective study of 200 patients with hepatic resection for HBV-related HCC, both conducted by Hung et al. [179, 180], patients with a high serum HBV viral load at the time of tumor resection showed a significantly higher recurrence rate compared with patients with a low viral load. Therefore, antiviral and antiinflammatory therapies after curative treatment may be crucial in preventing HCC recurrence and improving survival.

#### Interferon

A small RCT was performed to evaluate the safety and efficacy of 16 weeks of interferon α-2b therapy after hepatic resection in a group of patients with predominantly HBV-related HCC [181]. The RR of death after interferon treatment was 0.42 (95% CI 0.17–1.05, *p* = 0.06). Subset analysis showed that adjuvant interferon had no survival benefit for pTNM stage I/II tumors (5-year survival 90% in both groups, *p* = 0.91), but prevented early recurrence and improved the 5-year survival of patients with stage III/IVA tumors from 24 to 68% (*p* = 0.04). After this study, another similar RCT was conducted by Chen et al. [182]. A total of 268 patients were allocated randomly to receive either 53 weeks of adjuvant interferon α-2b treatment or observation alone. The primary endpoint of this study was RFS. The median RFS in the interferon α-2b and control arms was 42.2 and 48.6 months, respectively (*p* = 0.83). In this study, adjuvant interferon α-2b did not reduce the postoperative recurrence of HBV-related HCC. HCC recurrence after ablative treatment modalities is also common. Although patients who received medical ablation usually exhibit compensated hepatic functional status, the frequent recurrence of HCC after successful ablation contributes to short-term survival. A small RCT was conducted to evaluate the effectiveness of interferon therapy in preventing HCC recurrence after successful medical ablation therapy for primary tumors [183]. The cumulative HCC recurrence rate of the patients treated with interferon-α and the control group was 25 and 40% at the end of 1 year, and 47 and 90% at the end of 4 years, respectively (*p* = 0.01). Furthermore, this study also showed that the prevention of HCC recurrence using interferon-α was effective in HBV-related HCC [183].

#### Nucleos(t)ide analogs

A retrospective study was conducted to evaluate the efficacy with or without using nucleos(t)ide analogs in patients following curative treatments for HBV-related HCC [184]. Cumulative OS rates of HCC were significantly different between the two groups (*p* < 0.01), and cumulative RFS rates of HCC were also significantly different (*p* < 0.01). Yin et al. [185] also reported that nucleos(t)ide analogs improved not only liver function but recurrence and OS rates. In another study, improvements of liver function and OS rates were reported even if the recurrence rates were not significantly different between groups treated with and without nucleos(t)ide analogs [186, 187]. In addition, a large-scale nationwide cohort study was reported from the Taiwan National Health Insurance Research Database [188]. Among 100,938 newly diagnosed HCC patients, they identified 4569 HBV-related HCC patients who received curative liver resection for HCC. The risk of first tumor recurrence was compared between patients who did not (untreated cohort, *n* = 4051) and did (treated cohort, *n* = 518) take nucleos(t)ide analogs. The treated cohort had a higher prevalence of liver cirrhosis when compared with the untreated cohort (48.6 versus 38.7%, *p* < 0.001), but had a lower risk of HCC recurrence [*n* = 106 (20.5%) versus *n* = 1765 (43.6%), *p* < 0.001] and lower overall risk of death [*n* = 55 (10.6%) versus *n* = 1145 (28.3%), *p* < 0.001]. After adjusting for competing mortality, the treated cohort had a significantly lower 6-year HCC recurrence rate (45.6%; 95% CI 36.5–54.6% versus untreated, 54.6%; 95% CI 52.5–56.6%, *p* < 0.001). Six-year overall mortality was 29.0% (95% CI 20.0–38.0%) for the treated and 42.4% (95% CI 40.0–44.7%, *p* < 0.001) for the untreated cohort. On modified Cox regression analysis, administration of nucleos(t)ide analogs (HR 0.67; 95% CI 0.55–0.81, *p* < 0.001) was independently associated with a reduced risk of HCC recurrence. One study elucidated the superior choice of nucleos(t)ide analogs [189]. A total of 865 HBV-related HCC patients received antiviral treatment at diagnosis or immediately following surgery (adefovir 10 mg per day in 300 patients, entecavir 0.5 mg per day in 325 patients, and lamivudine 100 mg per day in 240 patients). The 1-, 2-, and 3-year resistance rates were 0.9, 1.8, and 2.5%, respectively, for the entecavir group; 3.0, 8.3, and 12.0%, respectively, for the adefovir group; and 21.7, 31.7, and 39.6%, respectively, for the lamivudine group. The 3-year RFS for the entecavir group also differed significantly compared with the adefovir and lamivudine groups (HR 0.810; 95% CI 0.656–0.999, *p* = 0.049 and HR 0.737; 95% CI 0.591–0.919, *p* < 0.01). A randomized, placebo-controlled trial by Jang et al. [190] also showed that preemptive lamivudine therapy in patients receiving transarterial chemoembolization (TACE) significantly reduced the incidence of HBV reactivation (*p* < 0.01), overall hepatitis (*p* = 0.02), and severe hepatitis (*p* = 0.035) due to HBV reactivation after repeated TACE. However, prevention of HCC by preemptive lamivudine therapy was not shown because of advanced stage of HCC in patients receiving TACE in that trial [190]. Further prospective RCTs using a larger number of patients are required to assess its role in tertiary prevention of HCC.

### Tertiary prevention of HCV-related HCC

HCC is characterized by very frequent recurrence even after successful initial treatments, either surgical resection or medical ablation, and the risk of recurrence remains high for many years. Recurrence is particularly frequent with HCV-related HCC, and a substantial proportion of recurrences, especially in the late phase, is thought to represent de novo, or multicentric, hepatocarcinogenesis [191–193].

#### Interferon

Antiviral therapy, such as interferon, might reduce the overall incidence of recurrence by preventing de novo carcinogenesis. Indeed, several small-sized RCTs, performed in Japan or Taiwan, showed that the incidence of recurrence was reduced in HCV-related HCC by interferon therapy subsequent to initial HCC treatment [194, 195]. Other RCTs, also performed in Japan or Taiwan, failed to find a significant delay in the first recurrence with interferon therapy, but the second or third recurrence was significantly reduced especially in sustained responders, and the OS was improved [196–198]. Another RCT in Italy did not detect effects of interferon therapy on early recurrence but did find an effect for late recurrence: after an interval of more than 2 years, the rate seemed to be reduced among interferon responders [199]. These data are compatible with the hypothesis that de novo carcinogenesis was prevented by successful antiviral therapy. On the other hand, three reports on long-term observation of recurrence after interferon therapy following HCC treatment showed that the recurrence rate in interferon-treated patients decreased over time, suggesting that the growth of residual microscopic tumors had been delayed by interferon (in fact, the two presumed mechanisms are not necessarily mutually exclusive) [200–202]. Most of these studies used interferon monotherapy and suffered from low sustained response rates because most patients had advanced fibrosis or cirrhosis. Preventive effects of interferon on HCC recurrence have yet to be reevaluated using current, more efficient protocols.

#### DAA therapy

DAA therapies are promising pan-genotypic agents used to eradicate HCV. However, there is no evidence that DAA therapy will prevent HCC recurrence. One study by Reig et al. [203] reported that eradication of HCV with DAAs led to an unexpected HCC recurrence in some cases, and others reported DAAs did not lead to an unexpected HCC recurrence [204–206], although there is no evidence that SVR after DAA therapy reduces the incidence of recurrence in HCC patients receiving curative treatments. Furthermore, prospective studies are needed.

### Other tertiary preventions of HCC

Microscopic, intrahepatic residual tumors, including intrahepatic metastases, are a possible cause of HCC recurrence. Theoretically, adjuvant chemotherapy may reduce or delay such recurrences, but few chemotherapeutic agents have been effective against HCC and many of them may be hepatotoxic.

#### Chemotherapy

Hasegawa et al. [207] reported RCT using oral administration of uracil-tegafur after curative hepatic resection but found no beneficial effects on recurrence and a possible adverse effect on OS. Bruix et al. [208] assessed the efficacy and safety of sorafenib versus placebo as adjuvant therapy in patients with HCC after surgical resection or local ablation. It was a double-blind, placebo-controlled study of patients with HCC with complete radiologic response after surgical resection (*n* = 900) or local ablation (*n* = 214) at 202 sites (hospitals and research centers) in 28 countries. At final analysis, 464 RFS events had occurred (270 in the placebo group and 194 in the sorafenib group). Median follow-up for RFS was 8.5 months in the sorafenib group and 8.4 months in the placebo group. There was no difference in median RFS between the two groups (33.3 versus 33.7 months, respectively; HR 0.940; 95% CI 0.780–1.134; one-sided *p* = 0.26) [208]. In 1996, Muto et al. [209] reported that administration of polyprenoic acid, an acyclic retinoid, reduced the recurrence of HCC in RCT. Updated, long-term data were published subsequently [210], postulating that the eradication of premalignant or latent malignant clones was the mechanism of action. However, in a large-scale RCT, the superiority of acyclic retinoid over placebo could not be validated, 600 mg per day was shown to be the optimal dose, and treatment may possibly reduce the recurrence of HCV-related HCC, particularly after 2 years [211]. The investigators concluded that administration of 600 mg per day of acyclic retinoid to patients with HCV-related HCC who have completed curative therapy might improve survival for those classified as having Child–Pugh class A disease, for whom liver function was relatively stable in subanalysis [212]. Other adjuvant treatments have not been shown to prolong RFS (Table 2).

**Table 2:** Adjuvant treatments preventing hepatocellular carcinoma recurrence

Refs.	Study design	Drug	Patients	Outcomes
Takami et al. [213]	RCT	Meloxicam	Meloxicam (*n* = 111) versus control (*n* = 113)	Negative. 3-year RFS 53.9% in meloxicam group versus 57.0% in controls
Habu et al. [214]	RCT	Menaquinone	Menaquinone (*n* = 21) versus control (*n* = 19)	Positive. Assessing only recurrence event: 9.5% in menaquinone group versus 47.4% in controls
Mizuta et al. [215]	RCT	Menatetrenone	Menatetrenone (*n* = 32) versus control (*n* = 29)	Positive. 24-month recurrence rate 39.0% in menatetrenone group versus 83.2% in controls and also assessing overall survival
Hotta et al. [216]	RCT	Menatetrenone	Menatetrenone (*n* = 21) versus control (*n* = 24)	Positive on recurrence event: 33.3% in menatetrenone group versus 50.0% in controls, but negative on cumulative recurrence rate
Yoshida et al. [217]	RCT	Menatetrenone	Menatetrenone (*n* = 367) versus controls (*n* = 181)	Second interim analysis indicated that vitamin K2 did not prevent disease occurrence or death, with HR of 1.150 (95% CI 0.843–1.570, *p* = 0.811)

## Diagnosis and surveillance

Imaging modalities

### Ultrasound (US) and contrast-enhanced ultrasound (CEUS)

#### Recommendations

1. Ultrasonography (US) is a screening test and not a diagnostic test for confirmation (B2).
2. Contrast-enhanced US (CEUS) is useful for characterization of US-detected liver nodules and is as sensitive as dynamic computed tomography (CT) or dynamic magnetic resonance imaging (MRI) in the diagnosis of HCC (B2).

As the prognosis of HCC depends largely on the stage at which the tumor is detected, detection of HCC early in its development is critical to improve the survival of affected patients [218–220]. Although ultrasonography (US) is the most widely used modality for HCC screening and surveillance, the reported sensitivity of surveillance US is in the range of 40–81% with specificity of 80–100% [221–225]. According to a recent meta-analysis study, among B-mode US, contrast-enhanced US (CEUS), contrast-enhanced (CT), and gadolinium-enhanced (MRI), B-mode US has the lowest sensitivity and positive predictive value (59.3, 77.4%) while the other three imaging modalities show similar pooled per-lesion sensitivity and positive predictive value (73.6–84.4%, 83.6–89.3%) [226]. Therefore, US is not advocated as a diagnostic test for confirmation due to overlapped imaging features of benign and malignant cirrhotic nodules on US.

Key alterations during hepatocarcinogenesis include angiogenesis, changes in cellularity, the transporters of hepatocytes, and decrease in the number and function of Kupffer cells [227]. Among them, hemodynamic alteration of the nodules, composed of increased arterial flow and decreased portal flow, is the most important change for the diagnosis of HCC [228–230]. However, B-mode US cannot demonstrate tumor vascularity, and color Doppler imaging and power Doppler imaging have low sensitivity for detecting the microflow in the nodules [231–235]. CEUS using microbubble contrast agents and low mechanical index (MI) contrast-specific imaging techniques has been proved to be useful for characterizing liver tumors [235, 236]. Moreover, as Sonazoid microbubbles are phagocytosed by Kupffer cells, Kupffer imaging can be achieved [237]. CEUS can provide superior sensitivity to detect arterial hypervascularity and better demonstration of rapid wash-out for non-HCC malignancy and very late wash-out of HCC compared with dynamic CT or dynamic MRI [238–240]. In addition, CEUS has several other advantages including relative inexpensiveness, no nephrotoxicity of the contrast agents, and no ionizing radiation. In general, CEUS shares many features with dynamic CT and dynamic MRI, but as they are purely intravascular, in cholangiocarcinoma a discordant enhancement pattern is observed on CEUS [236, 238, 241–243]. The AASLD removed CEUS from their guidelines in part because of the perceived possibility of false-positive HCC diagnosis in patients with intrahepatic cholangiocarcinoma [63, 242], but according to recent studies, wash-out time >55 s identified patients with HCC with the highest level of accuracy (92.7%) while wash-out time ≤55 s correctly identified the vast majority of non-HCC malignancies (diagnostic accuracy 98.3%) [244, 245]. In terms of diagnostic accuracy of CEUS for small HCC, recent meta-analysis studies demonstrated that pooled per-lesion sensitivity and positive predictive value of CEUS are similarly high (84.4 and 89.3%) compared with CT (73.6 and 85.8%) and MRI (77.5 and 83.6%), with better cost-effectiveness than CT or MRI [226, 246, 247]. A comparison of the diagnostic ability for hepatic nodules between CEUS using Sonazoid and contrast-enhanced CT showed that the sensitivity and accuracy were significantly higher for the former (95.4 and 94.7%) than the latter (85.2 and 82.3%) [248]. In real clinical practice, however, given that cirrhotic liver has a limited sonic window for whole-liver evaluation and that there is a strong need for CT or MRI for tumor staging, use of CEUS as a first-line diagnostic approach, albeit possible, may not be more cost-effective than CT or MRI. As of now, it is generally accepted that CEUS is a cost-effective second-line imaging modality for rapid diagnosis of HCC once the liver focal lesion is detected on US, although dynamic CT or dynamic MRI is the gold standard for characterization of small nodules at high risk for HCC in cirrhotic liver in Western guidelines [2, 249].

### CT and MRI

#### Recommendations

1. Dynamic CT, dynamic MRI, or gadolinium ethoxybenzyl diethylenetriamine pentaacetic acid (Gd-EOB-DTPA)-enhanced MRI is recommended as a first-line diagnostic tool for HCC when a screening test result is abnormal (A1).
2. Hallmark of HCC during dynamic CT scan or dynamic MRI is the presence of arterial enhancement, followed by wash-out of the tumor in the portal venous and/or delayed phases (A1).
3. HCC is diagnosed on the basis of imaging criteria in patients belonging to the high-risk group (chronic hepatitis B, chronic hepatitis C or cirrhosis) (A1).
4. The combined interpretation of dynamic and hepatobiliary phase of Gd-EOB-DTPA-enhanced MRI with diffusion-weighted imaging (DWI) can improve the diagnostic accuracy of MR imaging for the detection of HCC (B2).

Once a screening test result is abnormal or there is clinical suspicion of HCC, imaging plays a very important role for diagnosis and staging of this tumor [218, 250–252]. The radiological stage is used to inform clinical decision-making, optimize treatment strategies, and determine eligibility and priority for LT [252, 253]. As mentioned above, in addition to typical hemodynamic changes of HCC such as increased arterial flow and decreased portal flow, several pathologic changes can occur during development of HCC, including changes in cellularity, the transporters of hepatocytes such as organic anionic transporting polypeptides (OATP), and a decrease in the number and function of Kupffer cells [218, 227, 229, 254–257]. Accumulating data demonstrate that OATP8 expression level decreases during hepatocarcinogenesis prior to reduction in portal venous flow and prior to complete neoarterialization and to elevation of arterial flow, which may allow higher sensitivity for detection of malignant changes [257, 258]. The most reliable diagnostic tests for HCC diagnosis are quadruple-phase, multidetector CT (MDCT) and dynamic MRI including late hepatic arterial, portal venous, and delayed phase imaging at about 3–5 min after contrast administration [259–261]. Dynamic CT and dynamic MRI with extracellular gadolinium agents permit diagnosis and staging of HCC based mainly on assessment of vascularity [218, 251]. Presence of arterial enhancement followed by wash-out has sensitivity and specificity of 90 and >95%, respectively, and positive predictive value approximating 100% among the group having high risk for developing HCC, e.g., those with liver cirrhosis [262–265]. When extracellular agents are used, dynamic CT and dynamic MRI permit diagnosis and staging of HCC based mainly on assessment of vascularity, and the hallmark of HCC on CT or MRI is presence of arterial hyperenhancement (wash-in) followed by wash-out of the tumor in the portal venous and/or delayed phases [224, 266–268]. Sangiovanni et al. [269] also reported that the sensitivity of contrast-enhanced MDCT, and MRI using extracellular contrast medium for 1–2-cm HCCs was 44 and 44%, with 100% specificity. This may be explained by the fact that its diagnostic hallmark is often unseen in small HCCs (≤2 cm), resulting from incomplete neoangiogenesis [224]. According to several recent meta-analysis studies on the diagnostic accuracy of US, CT, and MRI [225, 226, 270, 271], the per-lesion sensitivity of MRI for nodular HCC of all sizes is 77–100%, while that of CT is 68–91%, and MRI showed at least equivalent or higher per-lesion sensitivity compared with MDCT and therefore could be the preferred imaging modality for diagnosis of HCCs [269, 272–275]. The per-lesion sensitivity, stratified by size, was 100% for both modalities for nodular HCCs > 2 cm, 44–47% (MRI) and 40–44% (CT) for 1–2 cm HCCs [269, 272, 276], and 29–43% (MRI) and 10–33% (CT) for HCCs < 1 cm [269, 273, 276]. To date, there are insufficient data regarding the specificity of the combined criteria of wash-in and wash-out appearance in subcentimeter cirrhotic nodules for HCC diagnosis on dynamic CT or dynamic MRI.

More recently, cell-specific contrast agents other than nonspecific extracellular gadolinium-based contrast media such as superparamagnetic iron oxide (SPIO) particles or in conjunction with gadolinium-based contrast agents (double contrast) or gadolinium ethoxybenzyl diethylenetriamine pentaacetic acid (Gd-EOB-DTPA) have been shown to be highly sensitive for detection of HCC, particularly for small tumors [218, 270, 277–285]. Several studies demonstrated that hepatobiliary contrast media, gadoxetate disodium (Gd-EOB-DTPA, Primovist, Bayer Healthcare, Berlin, Germany) and gadobenate dimeglumine (Gd-BOPTA, Multihance, Bracco, Milan, Italy), have higher overall sensitivity than dynamic CT or dynamic MRI using nonspecific gadolinium chelates [218, 270, 281–285]. A recent meta-analysis study demonstrated that Gd-EOB-DTPA-enhanced MRI showed significantly higher per-lesion sensitivity than MRI performed with other contrast agents (87 versus 74%) [270]. However, it should be noted that approximately 10–20% of HCCs may appear as iso- to hyperintense nodules on hepatobiliary phase (HBP) images [268, 286]. Despite the great advantage of Gd-EOB-DTPA-enhanced MRI for detection of liver malignancies, one possible pitfall of Gd-EOB-DTPA-enhanced MRI arises from absence of its equilibrium phase, which can show better wash-out of HCC than portal phase of dynamic CT or dynamic MRI. Indeed, hypointensity relative to the liver in the transitional phase (1–5 min) of Gd-EOB-DTPA-enhanced MRI may reflect hyperenhancement of liver parenchyma rather than deenhancement of a mass (“pseudo-wash-out”), thereby lowering the specificity for HCC diagnosis [252, 287]. Therefore, to maintain specificity, only portal venous phase “wash-out” should be used for a noninvasive HCC diagnosis, because malignant lesions other than HCCs, such as intrahepatic cholangiocarcinoma as well as hemangioma, can show hypointensity on the transitional phase and/or HBP [224, 288, 289].

Furthermore, diffusion-weighted imaging (DWI) may improve the diagnostic performance of MRI for small HCCs by demonstrating higher cellularity of HCC [290–292]. However, although hypointensity on the HBP and diffusion restriction could improve the sensitivity for the diagnosis of HCC, these findings are not specific to HCC and can be found in other hepatic tumors [224]. Other ancillary imaging features favor HCC diagnosis, including presence of intralesional fat, mild to modest hyperintensity on T2-weighted images [259, 293, 294], and morphologic findings such as intratumoral hemorrhage, fatty metamorphosis, and nodule-in-nodule architecture [252, 259]. However, great caution is still required when applying these ancillary imaging features for atypically enhancing cirrhotic nodules in order to retain high specificity, facing clinicians with the dilemma of balancing sensitivity and specificity [224].

Although CT hepatic arteriography (CTHA) and CT during arterial portography (CTAP) images have been used as the gold-standard diagnostic method for estimating the malignancy grade based on hemodynamic alteration, this has fallen out of favor in most practice settings except in some countries due to its invasiveness and high false-positive diagnosis rates [229, 295–297].

Hypovascular nodules associated with liver cirrhosis include low-grade dysplastic nodule (LGDN) or high-grade dysplastic nodules (HGDN), early HCCs, and well-differentiated HCCs [218, 255, 266, 298–302]. As there are significant overlaps in enhancement patterns on dynamic CT or dynamic MRI [295–297, 303, 304], the sensitivity of dynamic CT or dynamic MRI in detection of borderline nodules is quite low [301]. When detectable, most borderline lesions have a low–low–low, iso–low–low, or iso–iso–low enhancement pattern compared with adjacent background liver parenchyma on CT or MRI during the hepatic arterial, portal venous, and delayed phases [218, 301, 305, 306]. However, because expression of OATP8 decreases during hepatocarcinogenesis before complete neoarterialization, early HCCs may be more frequently visible on the HBP images of Gd-EOB-DTPA-enhanced MRI as hypointense nodules [289, 296, 297, 300, 307–313]. HBP hypointensity is a strong predictor of premalignancy or malignancy, and its presence favors HGDN or early HCC over LGDN or cirrhotic nodule [310, 314–316]. Although several imaging features are reported to be associated with interval progression to hypervascular HCCs, including large (>9–10 mm diameter) nodule size on initial imaging, nodule growth speed, hyperintensity on T2-weighted images or DWI, hyperintensity on pre-T1-weighted imaging, and intratumoral fat components [289, 315, 317, 318], it is quite challenging to differentiate early HCC from HGDN based on MRI findings [315]. More recently, when hypovascular nodules are detected by MDCT and MRI, the guidelines published by the (JSH) recommend use of CEUS using Sonazoid and Gd-EOB-DTPA-enhanced MRI [319]. The guidelines published by the JSH stated that hypovascular nodules that are hypointense in the hepatobiliary phase of Gd-EOB-DTPA-enhanced MRI and hypoechoic in the Kupffer phase of CEUS using Sonazoid can almost always be diagnosed as early HCC even without biopsy. However, it is highly likely that there may be some overlaps between early HCCs and HGDNs on both Gd-EOB-DTPA-enhanced MRI and CEUS using Sonazoid, so these noninvasive diagnostic criteria for hypovascular nodules need to be confirmed with further studies. As of now, those hypovascular nodules showing hypointensity on HBP of Gd-EOB-DTPA-enhanced MRI and decreased uptake in the Kupffer phase of CEUS using Sonazoid require biopsy and pathologic confirmation, as they possess high malignant or premalignant potential.

### Tumor markers

#### Recommendations

1. Alpha-fetoprotein (AFP) is not recommended as a confirmatory test in small HCC (B1).
2. The cut-off value of AFP should be set at 200 ng/mL for surveillance programs when used in combination with US (B2).
3. The cut-off value of AFP can be set at lower value in a population with hepatitis virus suppression or eradication (B2).

Tumor markers for HCC are used in diagnosis and treatment evaluation and during follow-up after treatment. The diagnostic performance of tumor markers is evaluated in terms of sensitivity, specificity, and likelihood ratios for positive and negative results (LR+/LR−) [320]. There is an inverse relationship between sensitivity and specificity according to cut-off values. Setting a lower cut-off value increases sensitivity and decreases specificity, and vice versa. A tumor marker with high LR+ is useful in confirming diagnosis, whereas a tumor marker with high LR− is useful in exclusive diagnosis. Those likelihood ratios also change according to cut-off values. The serum level of a tumor marker usually increases as the total tumor volume increases. This fact indicates that the sensitivity of a tumor marker essentially decreases as the target tumor size gets smaller when the cut-off value is fixed.

Since surveillance with alpha-fetoprotein (AFP) alone is only acceptable in a population-based setting and not recommended for high-risk population for HCC [321], the optimal cut-off value of AFP for surveillance should be determined on the premise that it is examined simultaneously with US. In such a situation, lower cut-off value increases the frequency of recall procedures and subsequent negative results and decreases the efficiency of the program.

Combination of two or more tumor markers may contribute to increased sensitivity without decreasing specificity when the correlation among them is small enough. However, to date the efficacy of adding another tumor maker to a surveillance program with US and AFP has not been fully assessed, especially in terms of cost-effectiveness.

### AFP

AFP has served as a diagnostic test for HCC since the 1970s, when most patients with HCC were diagnosed at advanced stage and with clinical symptoms [322]. Concentration higher than 500 ng/mL was diagnostic. However, the usefulness of AFP as a diagnostic test in small HCCs is limited. According to a systematic review, the sensitivity, specificity, and LR+ of AFP in HCC smaller than 5 cm in diameter ranged from 0.49 to 0.71, 0.49 to 0.86, and 1.28 to 4.03, respectively, with cut-off value of 20 ng/mL and from 0.04 to 0.31, 0.76 to 1.0, and 1.13 to 54.25, respectively, with cut-off value of 200 ng/mL [323]. In meta-analysis, AFP with cut-off value of 200 ng/mL showed a better combined LR+ than with that of 20 ng/mL (5.85 versus 2.45). The cut-off value of AFP should be set at 200 ng/mL instead of 20 ng/mL when used with US in a surveillance program, considering its efficiency.

It is well known that AFP levels increase in patients with active hepatitis or cirrhosis and without HCC, reflecting necroinflammation and regeneration; this fact is the major cause of its low specificity in high-risk population. On the other hand, AFP levels decrease according to decreased hepatitis activity by nucleos(t)ide analogs in chronic hepatitis B and by interferon-based treatments in chronic hepatitis C. In fact, increased sensitivity of AFP in those populations was reported, setting lower cut-off values [324, 325].

### Des-gamma-carboxyprothrombin (DCP)

Des-gamma-carboxyprothrombin (DCP), also known as prothrombin induced by vitamin K absence-II (PIVKA-II), is an abnormal prothrombin protein that is increased in the serum of HCC patients. Since the report by Liebman et al. [326], DCP has been recognized as not only a highly specific marker for HCC but also a predictor of prognosis of HCC patients [327, 328]. According to a systematic review, the sensitivity, specificity, and LR+ of DCP in HCC smaller than 5 cm in diameter ranged from 0.14 to 0.54, 0.95 to 0.99, and 6.86 to 29.7, respectively, with cut-off value of 40 mAU/mL and from 0.07 to 0.56, 0.72 to 1.0, and 3.56 to 13.0, respectively, with cut-off value of 100 mAU/mL [323]. In meta-analysis, DCP with cut-off value of 40 mAU/mL showed a better combined LR+ than with that of 100 mAU/mL (12.60 versus 4.91). According to a more recent systematic review, DCP showed better diagnostic performance than AFP in diagnosis of early HCC in terms of area under the receiver operating characteristic (ROC) curves (0.84 versus 0.68) [329]. However, funnel plot analysis suggested the presence of publication bias in DCP studies (*p* = 0.02). In fact, in a large-scale study enrolling 1377 patients with HCC and 355 with chronic hepatitis or cirrhosis, the diagnostic performance of DCP was inferior to that of AFP in terms of area under the ROC curves in small (<5 cm) HCC [330].

### *Lens culinaris* agglutinin-reactive fraction of AFP (AFP-L3)

AFP-L3 is a fucosylated variant of AFP that reacts with *Lens culinaris* agglutinin A and can differentiate an increase in AFP due to HCC from that in patients with benign liver disease [331–333]. According to a systematic review, the sensitivity, specificity, and LR+ of AFP-L3 in HCC smaller than 5 cm in diameter ranged from 0.22 to 0.33, 0.93 to 0.94, and 4.63 to 30.8, respectively, with cut-off value of 10% and from 0.21 to 0.49, 0.94 to 1.0, and 8.06 to 45.1, respectively, with cut-off value of 15% [323]. In meta-analysis, AFP-L3 with cut-off value of 15% showed better combined LR+ than with that of 10% (13.1 versus 4.89). One of the major drawbacks of AFP-L3 was that it could not be measured when the AFP value was less than 10 ng/mL. Recently, a highly sensitive assay system was developed which enables AFP-L3 measurement in the range of AFP less than 10 ng/mL [334].

### Glypican-3 (GPC3)

Glypican-3 (GPC3) is a heparan sulfate proteoglycan anchored to the plasma membrane. It has been reported that GPC3 messenger RNA levels are increased in HCC [335, 336]. Whereas the role of GPC3 in immunohistochemical staining was established [337], the reported diagnostic performance of serum GPC3 was inconsistent, mainly due to heterogeneous and unestablished assay system [338].

### Other tumor markers

Various tumor markers have been proposed including Golgi protein 73 (GP73) [339], osteopontin [340], circulating cell free DNA [341], and microRNAs [342]. However, none of them were introduced into daily practice, mainly due to significant heterogeneity in reports and lack of profitability regarding cost-effectiveness.

### Combination of tumor markers

Simultaneous measurement of tumor markers enables improved sensitivity without deteriorating specificity when they have weak association. The sensitivity, specificity, and LR+ of AFP and DCP in small HCC were reported to be 0.48, 0.99, and 48 with cut-off value of 200 ng/mL for AFP and 40 mAU/mL for DCP [343]. A more recent systematic review reported that the area under the ROC curve was not improved by the combination of DCP and AFP (0.83) compared with DCP alone (0.84) [329].

### Diagnostic algorithm

#### Recommendations

1. Typical HCC can be diagnosed by imaging, regardless of its size, if a typical vascular pattern (i.e., arterial enhancement with portal venous wash-out) is obtained on dynamic CT, dynamic MRI, or CEUS (A1).
2. Nodular lesions that show an atypical imaging pattern (e.g., iso- or hypovascular in the arterial phase or arterial hypervascularity alone without portal venous wash-out) should undergo further examination (A1).
3. Gd-EOB-DTPA-enhanced MRI can detect the earliest initial change of HCC, including HGDN, and early HCC (B1).

This section of the guidelines is markedly revised from the APASL 2010 guidelines [4]. Various studies have verified the usefulness of Gd-EOB-DTPA-enhanced MRI for diagnosis of HCC [268, 286, 296, 297, 300, 311–313, 317, 318, 344–363], although this method is not yet included in the AALSD or European Association for Study of the Liver (EASL) guidelines [2, 63, 364, 365]. Only the updated APASL diagnostic algorithm includes Gd-EOB-DTPA-enhanced MRI as a first-line diagnostic tool for HCC, similar to the JSH-LCSGJ guideline [366].

Many institutions use US to screen for HCC, followed by dynamic CT or dynamic MRI for subsequent examinations. When a lesion is intensely enhanced in the arterial phase and shows hypoenhancement in the equilibrium phase by dynamic CT or transitional phase by Gd-EOB-DTPA-enhanced MRI, a diagnosis of HCC is unproblematic; however, benign hypervascular lesions (such as high-flow-type hemangioma), cholangiocarcinoma or combined HCC must be ruled out. When the hepatobiliary phase of Gd-EOB-DTPA-enhanced MRI or the Kupffer phase of CEUS using Sonazoid confirms a defect in these hypervascular nodules, the lesion is diagnosed as HCC.

When a lesion shows low attenuation in the equilibrium phase of dynamic CT, even though it is not intensely enhanced during the early arterial phase, it is possible that a more sensitive tool may diagnose it as hypervascular HCC; thus, either Gd-EOB-DTPA-enhanced MRI or CEUS is necessary. Gd-EOB-DTPA-enhanced MRI is useful for differentiating HCC (even early HCC) from a dysplastic nodule (DN) [299, 309, 367, 368].

Among the nodular lesions associated with liver cirrhosis, LGDN and HGDN (both of which are considered to be precancerous lesions), early HCC, and nodule-in-nodule liver cancer are regarded as nonhypervascular [299, 309, 367, 368]. The most sensitive modalities that can objectively depict the early carcinogenic process are (1) Gd-EOB-DTPA-enhanced MRI, followed by (2) CTAP/CTHA [369, 370], and (3) CEUS [237, 362, 371]. Portal blood flow may be maintained in some cases of DN and early HCC, but is reduced in other nodules, although arterial blood flow in cases of DN and early HCC will not have increased yet.

The hepatobiliary phase of Gd-EOB-DTPA-enhanced MRI can detect the earliest initial changes suggestive of HCC. The second earliest initial carcinogenic changes are detected by CTAP and the third earliest by CTHA or CEUS (an increase in intranodular arterial blood flow). However, because CTHA and CTAP are invasive tests, they are only performed in a few countries. Indeed, they are not common in the majority of countries in the Asia–Pacific region. Since Gd-EOB-DTPA-enhanced MRI can identify initial carcinogenic changes earlier than CTHA and CTAP [258, 357, 372], the latter have been almost completely replaced by Gd-EOB-DTPA-enhanced MRI. Hypervascular lesions depicted as nodule-in-nodule or as entire hypervascular nodules can be interpreted as advanced cancer, even though they are very small (<2 cm).

Dynamic CT and Gd-EOB-DTPA-enhanced MRI show high sensitivity for arterial blood flow, but cannot detect arterial vascularity in some nodules (detection depends on acquisition timing, tumor location, and liver function), even though lesions appear hypervascular on CEUS. Nodules showing intense enhancement on dynamic CT and Gd-EOB-DTPA-enhanced MRI are assumed to exhibit high intensity on T2-weighted images and DWIs of MRI.

It is recommended that institutions specializing in liver cancer use Gd-EOB-DTPA-enhanced MRI rather than dynamic CT, even when no tumor is detected on US. Institutions that cannot perform Gd-EOB-DTPA-enhanced MRI as the first-line modality may use dynamic CT as a first screening/diagnostic step, even when no nodule is evident on US; however, it is absolutely essential that Gd-EOB-DTPA-enhanced MRI or CEUS be performed when dynamic CT does not identify hallmarks of HCC (i.e., arterial enhancement with venous wash-out) in the detected nodule.

**Fig. 1:**
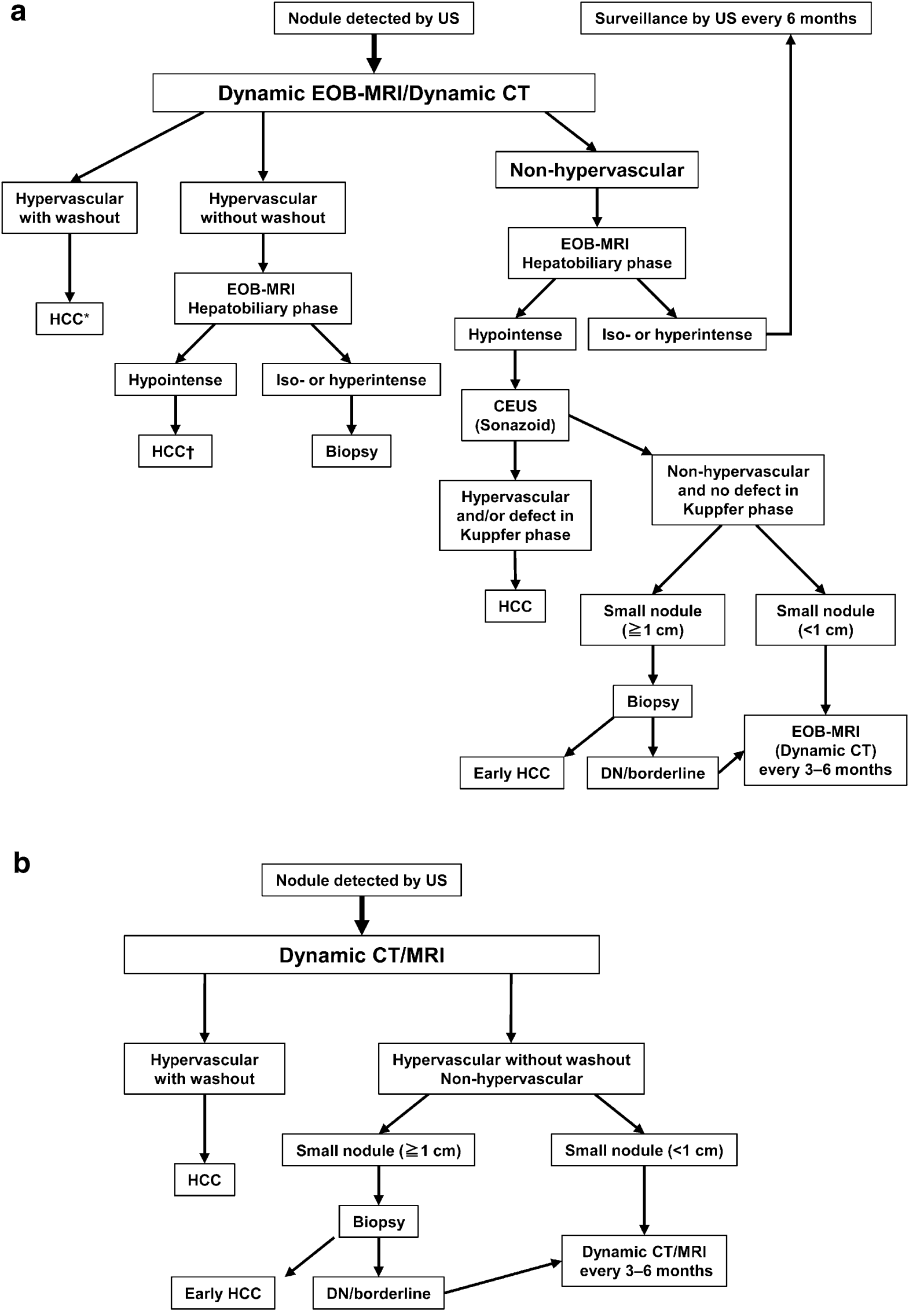
Diagnostic algorithm for hepatocellular carcinoma using multiple modalities (**a**) and only dynamic CT/MRI (**b**) (APASL 2016). *Cavernous hemangioma sometimes shows hypointensity on the equilibrium (transitional) phase of dynamic Gd-EOB-DTPA MRI (pseudo-wash-out). It should be excluded by further MRI sequences and/or other imaging modalities. †Cavernous hemangioma usually shows hypointensity on the hepatobiliary phase of Gd-EOB-DTPA MRI. It should be excluded by other MRI sequences and/or other imaging modalities

If Gd-EOB-DTPA-enhanced MRI (or dynamic CT) identifies a hypervascular nodule with venous wash-out, a definitive diagnosis of HCC can be made. If Gd-EOB-DTPA-enhanced MRI (or dynamic CT) shows a hypervascular nodule without venous wash-out, a diagnosis of HCC can be made if the nodule shows hypointensity in the hepatobiliary phase of Gd-EOB-DTPA-enhanced MRI. Also, in this case, another modality or MRI sequence should be used to rule out high-flow-type hemangioma, because the latter can exhibit characteristics similar to HCC. If the hepatobiliary phase of Gd-EOB-DTPA-enhanced MRI identifies the nodule as isointense or hyperintense, biopsy is necessary to confirm the diagnosis (Fig. 1a).

Isointense or hyperintense nonhypervascular nodules in the hepatobiliary phase of Gd-EOB-DTPA-enhanced MRI can enter the routine surveillance protocol. However, nonhypervascular hypointense nodules have high potential for malignant transformation [296, 297, 311–313, 317, 318, 345, 346, 351, 355, 356, 373–379], therefore CEUS study using Sonazoid is highly recommended. HCC can be correctly diagnosed by CEUS if hypervascularity and/or a defect in the Kupffer phase is observed. Even when a nodule is hypovascular on CEUS with an evident defect in the Kupffer phase, a finding of hypointensity in the hepatobiliary phase of Gd-EOB-DTPA-enhanced MRI is highly suggestive of malignancy [300]. Accordingly, biopsy is recommended for nodules 1.0 cm or larger to make a differential diagnosis between early HCC and a DN. If a nodule is diagnosed as a DN or a borderline lesion, intensive follow-up (every 3–6 months) with Gd-EOB-DTPA-enhanced MRI (or dynamic CT) is recommended. Intensive follow-up is also recommended for nodules smaller than 1.0 cm (Fig. 1a). In field practice, multiple imaging modalities are not available at all institutions. Thus, a diagnostic algorithm based on using only dynamic CT (or MRI) is shown in Fig. 1b.

### Surveillance

#### Recommendations

1. Surveillance for HCC should be undertaken in high-risk groups of patients and is recommended (B2). The high-risk groups of patients for whom a surveillance strategy is recommended are described in Table 3.

**Table 3:** Groups where HCC surveillance is recommended

	HCC risk (per year)
Cirrhotic hepatitis patients	
HBV	3–5%
HCV	2–7%
NASH	2–4%
Genetic hemochromatosis	Unknown, but probably >1.5%
Primary biliary cirrhosis	2–3%
Alpha 1 antitrypsin (A1AT) deficiency	Unknown, but probably >1.5%
Autoimmune hepatitis	
Other etiologies	Unknown
Chronic HBV carriers	
Noncirrhotic (HBsAg positive)	
Asian females >50 years	0.3–0.6%
Asian males >40 years	0.4–0.6%
Africans aged >20 years	NA
History of HCC in the family	NA
2. Measurement of AFP alone is not recommended for routine surveillance of HCC (A1).
3. The combination of US and serum AFP measurement performed biannually should be used as a surveillance strategy for HCC (B2).

Surveillance is continuous monitoring for disease occurrence and includes application of a diagnostic test in subjects who are predisposed to develop a given disease. The primary motive of a surveillance strategy is to achieve a reduction in disease-related mortality through prompt diagnosis (stage migration), which could, in turn, increase the cost-effectiveness and applicability of certain curative therapies. To consider an intervention effective, it must result in an increase in longevity of approximately 90 days, and if this goal can be attained at a cost of less than approximately US $50,000 per year of life gained, it can be deemed cost-effective [380].

### Which modality is to be used for surveillance?

Tests that are widely available include tumor markers, such as AFP, and various imaging techniques, including US, CT, and MRI of the abdomen.

#### US

US is widely used for surveillance of HCC; its widespread popularity is due to its potential advantages of being noninvasive, an absence of risks associated with the procedure, and good acceptance by patients at a relatively moderate cost. A meta-analysis that included 19 studies showed US to be less effective in detecting early-stage HCC (demonstrating sensitivity of only 63%). However, it could detect the vast majority of HCCs before the disease would present clinically (depicting pooled sensitivity of approximately 94%) [223]. In a study by Sato et al. [381] including 1431 patients with chronic HCV, US-based surveillance performed by trained operators resulted in early detection of HCC with average tumor size of 1.6 ± 0.6 cm and only 1.4% of cases exceeded tumor size of 30 mm. Thus, it was suggested that US-based surveillance performed biannually was adequate for early detection of HCC at size smaller than 3 cm [381]. The performance of US in an HCC surveillance strategy strongly depends on the quality of the equipment and the expertise of the performing operator. Thus, special training is warranted for ultrasonographers.

#### CT

Existing evidence does not support routine use of CT scan as part of the surveillance strategy for HCC. Patients with a ≥1 cm nodule in the liver are recommended to undergo a contrast-enhanced CT scan of the abdomen as a confirmatory test for the diagnosis of HCC, including unenhanced, arterial, venous, and delayed phase imaging. A randomized trial in 2013 examined 163 patients who had compensated cirrhosis. These patients were tested with either annual CT plus biannual AFP measurements or biannual US plus serum AFP measurements. The combination of biannual US and AFP was marginally more sensitive at detecting HCC compared with annual CT (sensitivity and specificity of 71.4 and 97.5%, respectively, versus 66.7 and 94.4%, respectively). This approach was deemed more cost-effective as well [222].

#### MRI

Similar to the recommendations made for CT scanning of the abdomen, no existing evidence is available to recommend use of abdominal MRI as part of a routine surveillance strategy for detecting HCC. For patients with a ≥1 cm nodule in the liver, dynamic imaging, such as contrast-enhanced MRI of the abdomen, is often needed as a confirmatory test.

#### AFP and other serum markers

Measurement of serum AFP levels is a commonly used strategy for surveillance of HCC because it is widely available, inexpensive, and easy to perform. However, AFP has suboptimal performance as a serological test for surveillance of HCC because it depicts fluctuating levels in patients with cirrhosis with a flare of HCV or HBV infection, in exacerbations of the underlying liver disease, or with the occurrence of HCC [382]. Abnormal serum AFP levels can be detected in only a meager proportion of early-stage HCC tumors (10–20%), which has been correlated now with a particular subtype of HCC depicting an aggressive behavior (S2 class, EpCAM positive) [383]. AFP level of 10.7 ng/ml showed the best combination of specificity (78.1%) and sensitivity (77.2%), a cutoff that approaches the routine limits of normalcy [384].

Other serum markers, such as (DCP), α-fucosidase, AFP-L3%, and GPC3, are used predominantly in the diagnostic rather than surveillance setting. The presence of elevated AFP-L3% is correlated with an HCC tumor with shorter doubling time, and raised serum DCP levels might be indicative of microinvasion [327, 385]. The HALT-C trial studied the AFP and DCP levels of 39 patients with HCC at diagnosis and 1 year before diagnosis. Neither test alone, nor the combination of the two, was adequate for HCC surveillance because the sensitivity of these two markers was very low when they were used either alone or in combination for the strategy to be considered efficacious and cost-effective in detecting HCC at an early stage [386]. Thus, at present, other than AFP, none of these markers can be recommended routinely as part of a surveillance strategy in patients at risk for HCC [327, 387].

#### Combination of imaging and serum markers

Conflicting results have been obtained in studies regarding combination of imaging modalities with serum biomarkers for surveillance of HCC.

The pooled data of a meta-analysis that included 19 studies revealed the combination of US and serum AFP measurement versus US alone to be less specific, no better at detecting subclinical and early-stage HCC, and also not cost-effective. Although the combination of US and serum AFP resulted in marginally increased sensitivity of 69% compared with 63% for US, this result was not statistically significant [209].

In contrast, a recent study demonstrated that serum AFP at cut-off of 20 ng/ml had specificity and sensitivity of 93.3 and 52.9%, respectively, whereas US had specificity and sensitivity of 74.2 and 92.0%, respectively. A combination of US and AFP demonstrated specificity and sensitivity of 68.3 and 99.2%, respectively. It was shown that, when using a cutoff level at 20 ng/ml and AFP level increase of ≥2 times from its nadir in the past 12 months, the combination of AFP and US depicted improved specificity of 71.5% and sensitivity of 99.2% [388].

The benefit of surveillance was demonstrated in a subset of patients with chronic HBV by Zhang et al. [389], in which biannual US and serum AFP measurement decreased mortality from HCC by 37%. Compliance with scheduled tests was depicted to be approximately 58.2%.

Several reports indicated the cost-effectiveness of HCC surveillance, and that US combined with AFP has been shown to increase quality-adjusted life years in patients who suffered from HCC, especially those who underwent resection or transplantation [390, 391]. The cost-effectiveness of HCC surveillance depends on the potential of receiving curative therapy in high-risk patients. Thus, if patients are ineligible for treatments due to severe liver disease or other comorbidities, HCC surveillance is not necessary.

### Surveillance interval

The surveillance interval should depend on the median tumor doubling time, which in HCC is demonstrated to be 80–117 days. Thus, a 6-month surveillance interval seems to be a reasonable choice. A meta-analysis has demonstrated that the pooled sensitivity of a US-based 6-month surveillance strategy drops to 50 from 70% for an annual program [223]. A study by Anderson et al. [392] demonstrated that semiannual US surveillance for HCC in cirrhotic patients improves clinical outcomes at a reasonable cost.

In a RCT that enrolled patients with compensated cirrhosis, no significant difference was documented in the rate of HCC detection by using an US-based surveillance strategy every 3 or 6 months [393]. Thus, in the light of current evidence, biannual US with AFP-based surveillance seems appropriate and is currently recommended.

### Who should be screened and who should not be screened?

The economic scenario in each country dictates the threshold at which a surveillance program can be considered cost-effective. Patients who have liver cirrhosis and those who have chronic HBV infection (even in the absence of cirrhosis) constitute the high-risk group (Table 3) [394].

#### Cirrhotic patients

Studies depicting cost-effectiveness suggest that an incidence of HCC of ≥1.5% per year would require implementing a surveillance strategy in patients with cirrhosis [395], which would be irrespective of the etiology involved [396, 397]. The presence of late-stage decompensated cirrhosis (Child–Pugh class C) prohibits use of potentially curative therapies, thus implementing surveillance strategies may not be a cost-effective approach in this subset of patients [398]. Cost-effectiveness of HCC surveillance depends on the potential of receiving curative therapy in high-risk patients. Thus, if patients are ineligible for treatments due to severe liver disease or other comorbidities, HCC surveillance is not necessary. An exception to this is patients who are on a wait list for liver transplantation, who should undergo screening for HCC regardless of their liver functional status, because detecting tumors that exceed the conventional criteria may help formulate priority policies for liver transplantation. A recent Danish nationwide cohort study of patients suffering from alcohol-related cirrhosis of the liver demonstrated that the 5-year cumulative risk of HCC was only 1.0% [399]. Thus, it was suggested that a surveillance strategy in this subset of patients might not prove to be cost-effective. However, further studies are needed to verify these findings.

#### Noncirrhotic patients

Patients with chronic HBV infection are also prone to HCC development in the noncirrhotic stage. The cut-off for the annual incidence of HCC is still ill defined in this subset of patients, although opinions from expert groups suggest that surveillance strategies are needed if the incidence of HCC is at least 0.2% per year [63]. The incidence of HCC developing in adult African or Asian active chronic HBV carriers or those having a history of HCC in the family exceeds this value, and Asian patients having high HBV-DNA levels (>10,000 copies/mL) in serum are linked to a yearly risk of more than 0.2%/year [127].

A recent study by Lok et al. [400] showed that HCC can occur in noncirrhotic patients with chronic HCV who suffer advanced fibrosis (METAVIR F3). Because the transition to cirrhosis from advanced fibrosis cannot be determined accurately, patients with chronic HCV with bridging fibrosis can be considered for surveillance; however, further data are needed before making this recommendation. Noninvasive methods to ascertain liver fibrosis, such as transient elastography (TE), appear to be novel tools to stratify patients at different HCC risks [401]. On the other hand, HCV-infected patients without cirrhosis remain at risk for HCC even after achieving SVR. Fibrotic stage (F2 or 3), old age, gamma-glutamyl transferase (γGT) levels, and DM carry high risk of HCC occurrence in noncirrhotic patients, and these patients should be followed carefully for HCC after SVR [402, 403].

Patients with NAFLD who do not have underlying cirrhosis might also benefit from surveillance strategies, because emerging evidence suggests an increased risk of HCC development in this subset of patients [404]; however, more data on this aspect are needed before this strategy is recommended routinely [63]. Groups for whom HCC surveillance is uncertain are shown in Table 4.

**Table 4:** Groups in which HCC surveillance is uncertain

Patient group	HCC risk (per year)
Chronic hepatitis C-induced advanced fibrosis	<1.5%
Chronic hepatitis B carriers younger than 50 years (females) or 40 years (males)	<0.2%
NAFLD, noncirrhotic stage	<1.5%

#### Treated chronic viral hepatitis

Patients who achieve sustained HBV-DNA suppression or HBeAg seroconversion in chronic HBV and SVR in chronic HCV have increased; however, those treatments do not eliminate the risk of HCC completely [405, 406]. Thus, surveillance can be offered to treated patients with chronic HCV who have advanced fibrosis or cirrhosis even after achieving SVR and also to patients with chronic HBV who remain at risk of HCC due to various baseline factors.

## Treatments

### Liver resection (LR) and liver transplantation (LT)

#### Recommendations

1. Liver resection (LR) is a first-line curative treatment for HCC among Child–Pugh class A patients when resectability is confirmed in terms of tumor burden and liver functional reserve by multidisciplinary evaluation (B2).
2. Liver transplantation (LT) provides the best curative treatment for all HCC patients from an oncologic point of view, and is recommended as a first-line treatment for HCC among Child–Pugh class B and C patients, if the liver graft is available (A1).
3. For cirrhotic Child–Pugh class A patients with HCC, resectability should be discussed in a multidisciplinary team, and LT may be a second-line treatment in a salvage fashion (B2).

The optimal surgical strategy for HCC has been controversial so far, as indicated by the great difference in the indication for liver resection (LR) and LT for HCC among major algorithms worldwide [407]. When considering LR for HCC, the extent of radical resection to remove the tumor, as well as the functional reserve of the diseased liver and the volume of the future liver remnant, must be taken into account. LT is now an established surgical treatment for HCC patients. In contrast to LR, there is no restriction for the indication of LT, at least in terms of liver function, and LT, which could potentially cure both the diseased liver and HCC, is superior to any other conventional therapeutic options from an oncologic point of view. It is now a matter of debate how best to select those to be offered LR or LT among HCC patients [408]. This section summarizes the current opinions regarding LR and LT for HCC.

### LR

Recent advances in surgical technique and postoperative management have made LR safe even for those with cirrhosis; however, there is still no consensus regarding the tumor burden and the liver functional reserve suitable for surgical removal with adequate survival. Indeed, in the current most popular guidelines, surgery is restricted to those patients in the very early or early stages of disease [Barcelona-Clinic Liver Cancer (BCLC) score 0–A] [2, 409]. However, LR in the real world is completely different from the BCLC recommendations, as demonstrated in the recent multicenter study reporting that 50% of patients with intermediate or advanced HCC are treated routinely with surgery in tertiary referral centers worldwide [410]. Thus, it seems difficult to set clear indication of LR for HCC at present, and LR should at least be considered in a multidisciplinary setting as a potentially curative therapy for not only patients with BCLC stage 0–A, but also patients with BCLC stages B and C. At present, however, the AASLD and EASL guidelines [2, 63], following the BCLC recommendations, set narrower indication for LR. LR is only recommended for those with single nodule and Child–Pugh class A without evidence of portal hypertension.

In contrast, LR is indicated for more progressed HCC in terms of tumor burden and for more diseased patients in terms of liver function in the treatment algorithms of Asian countries [411]. Firstly, in terms of tumor burden: The Japanese treatment algorithm recommends LR for those with single HCC (any size, regardless of macrovascular invasion) and those with multiple nodules within 3 in number (any size) [366, 412, 413]. The Hong Kong treatment algorithm recommends LR for those with early tumor (≤5 cm, ≤3 tumor nodules, no intrahepatic venous invasion) and intermediate tumor ([1] ≤5 cm, either >3 tumor nodules or with intrahepatic venous invasion, or [2] >5 cm, 3 tumor nodules, and no intrahepatic venous invasion) [414]. The Korean treatment algorithm adopts wider indication of resection for HCC in which LR is allowed for those with curatively treatable disease (no limit regarding tumor burden) [415]. Secondly, in terms of liver functional reserve: The Japanese treatment algorithm recommends LR for those with Liver Damage A and B [412]. The Hong Kong treatment algorithm recommends LR for those with Child–Pugh class A and B early tumor and those with Child–Pugh class A intermediate tumor [414].

### LT

LT is the only treatment that offers the real chance of a cure for both HCC and the underlying liver cirrhosis; the shortage of liver grafts and the possibility of tumor recurrence, however, are strong limiting factors. To minimize HCC recurrence, the Milan criteria are now accepted as the gold-standard patient selection criteria in terms of tumor burden: solitary HCC less than 5 cm in diameter or within 3 nodules less than 3 cm in diameter, and without radiological evidence of vascular invasion or distant metastasis [416]. The most widely accepted criteria for the expansion of Milan are the University of California, San Francisco (UCSF) criteria: solitary tumor ≤65 mm in diameter, or 2–3 tumors, each with diameter ≤45 mm and total tumor diameter ≤80 mm, and without radiological evidence of vascular invasion or distant metastasis [417]. While it is widely accepted that the Milan criteria are too strict in terms of posttransplant recurrence rate and could definitely be expanded to some extent without impairing patient outcome, one must always be aware that any kind of expansion in tumor size or number includes the potential to worsen the posttransplant survival in patients with HCC [418]. The “metroticket paradigm” well describes this principle: the longer the distance beyond the conventional indication criteria with more aggressive tumor burden, the higher the price in terms of postoperative impairment in survival. Excessive expansion of inclusion criteria will result in a significant increase in organ demand, with a consequent increase in waiting time and a deterioration of OS among patients with HCC as a whole in the corresponding region [419]. Moreover, the allocation system should take into account how much the extension of criteria for HCC patients will negatively influence the wait list of patients without HCC. According to studies based on the US transplant registry using Markov models, patients beyond the Milan criteria would need to achieve 5-year survival of above 60% to prevent a substantial decrease in the life-years available to the entire population of candidates for LT [420].

The Milan criteria are also standard indication criteria for LT for HCC patients in Asian countries. However, in Asia where living-donor liver transplantation (LDLT) is the mainstay for LT, things are somewhat different from region to region [421]. Unlike deceased-donor LT, LDLT is not limited by the restrictions imposed by the nationwide allocation system, and the indication for LDLT in patients with HCC often depends on institutional or case-by-case considerations, balancing the burden on the donor, the operative risk, and the OS benefit for the recipient. Caution should be paid to the possible increased recurrence rate in LDLT when compared with deceased-donor LT [422], while reports of this issue seem conflicting [423]. In Japan, each center has developed institutional expansion criteria, while National Insurance covers only those within the Milan criteria. In Taiwan and Hong Kong, the UCSF criteria [417] are adopted. In Mainland China, Hangzhou or Chengdu criteria are used with satisfactory outcome [424]. In Korea, the UCSF or Milan criteria are basically used, but LDLT can be offered for any HCC without distant metastasis under National Insurance coverage. In conclusion, the Milan criteria are still the gold-standard criteria of LT for HCC patients worldwide, and seem best to be included in the treatment algorithm for HCC to set the tumor burden limitation.

The indication of LT for HCC in terms of liver functional reserve is based on the model for end-stage liver disease (MELD) score with additional points in Western countries [418]. Consequently, LT can be offered for those with Child–Pugh class A as shown in the BCLC algorithm, if they satisfy the Milan criteria [425]. In contrast, in Asian countries, where liver grafts are extremely scarce, LT is recommended for those with decompensated liver cirrhosis (Child–Pugh class B and C) in patients with HCC as well as in those with other diseases.

### LR versus LT

LT is definitely superior to LR or other locoregional treatments from the oncologic viewpoint, since it enables the widest possible resection margins and completely removes the diseased liver at risk of developing HCC. Considering that 5-year survival after LR for HCC among those with Child–Pugh class B is around 60% at a maximum [426, 427], LT should be recommended for such patients, if the graft is available. On the contrary, there is ongoing controversy regarding the indication of LR and LT for HCC among those with Child–Pugh class A liver dysfunction [428–431]. As mentioned above, LT is recommended as a primary treatment for HCC among those with Child–Pugh class A with evidence of portal hypertension in Western countries; however, given the shortage of liver grafts, the selection of patients who can achieve a comparable outcome by LR is a matter of debate [429, 432, 433]. Chapman et al. [434] reported significantly worse patient survival and RFS of LR compared with LT among noncirrhotic patients with HCC within the Milan criteria. Similarly, Adam et al. [435] reported worse outcomes of LR against LT among those with solitary HCC with diameter less than 5 cm. The significantly impaired RFS of LR was observed even for those with solitary HCC less than 3 cm in diameter. On the contrary, Vitale et al. [436] found that LR achieved better patient survival regardless of tumor stage provided that the patient’s MELD score was less than 10. According to the meta-analysis performed by Proneth et al. [437], resectable HCC should primarily be resected as a good alternative to LT when both LR and LT seem feasible, although the data collected for the meta-analysis were of low quality of evidence. Some European authors reported that salvage LT following LR may have poorer outcomes than upfront LT [438, 439], although those are retrospective single-center observational studies. LR versus LT for those initially admissible for both treatments should be investigated by well-designed prospective study. In addition, one should always be aware that intention-to-treat analysis, not just survival from operation, should be considered when comparing LT and LR.

In contrast, in Asian countries where locoregional treatments are the mainstay strategy for HCC, LT is not recommended for Child–Pugh class A patients [411], and LR achieved 5-year survival rate of around 60% among Child–Pugh class A recipients even with portal hypertension [440], and when restricted to Child–Pugh class A patients within the Milan criteria, the 5-year survival rate reaches above 70% [441, 442]. Given the absolute scarcity of liver grafts and excellent locoregional treatment strategies in Asian countries, Child–Pugh class A, noncirrhotic patients with HCC should firstly undergo LR rather than LT if both are feasible, and the resectability of HCC should be evaluated in a multidisciplinary fashion for Child–Pugh class A, cirrhotic patients. Several methods for estimation of liver functional reserve, such as indocyanine green retention rate at 15 min (ICG-15), 99mTc-galactosyl human serum albumin (GSA) scintigraphy, 13C-methacetin breath test (LiMAx), MELD score, serum albumin-bilirubin (ALBI) grade, aspartate transaminase-to-platelet ratio index (APRI), and FibroScan may be helpful for further stratification among Child–Pugh class A patients to elucidate better candidates for LR (or LT).

## Decisions on resectability of HCC

### The perspective of surgeons

While discussing the resectability of HCC, both technical and oncological aspects should be taken into consideration, as for the case of colorectal liver metastases. Satisfactory long-term prognosis is required to justify surgical resection, even if a tumor is technically and safely resectable. However, it is quite difficult to define “satisfactory prognosis,” because various points need to be considered, including social, ethical, economic, and emotional issues. Thus, in this section, we focus on the technical aspects related to the resectability of HCC.

In general, the surgical indications for HCC are decided not only according to the conditions of the tumor, but also according to the liver function, because HCC is frequently associated with liver dysfunction or cirrhosis caused by viral hepatitis, steatohepatitis, alcohol abuse, etc. Extensive resection of noncancerous liver parenchyma, which is a risk factor for fatal postoperative liver failure, should be avoided as much as possible. To prevent postoperative liver failure, accurate preoperative estimation of both the liver functional reserve and liver volume to be resected is essential.

There are several methods available to estimate the liver functional reserve, such as determination of the Child–Pugh score, the MELD score, determination of the hepatic venous pressure gradient, 99mTc-galactosyl serum albumin liver scintigraphy, and measurement of the ICG R15. Although, in Western countries, determination of the Child–Pugh score is the standard method, it provides too rough an estimate to allow accurate quantitative evaluation of the liver functional reserve or accurate prediction of the surgical risk in patients with liver dysfunction. On the other hand, in Asian countries, the ICG R15 value is regarded as an important parameter to estimate the liver function and tolerable resection volume. Especially in Japan, the so-called Makuuchi’s criteria [443], which include ICG R15, have been widely applied to determine the surgical indications and surgical procedures for HCC. Several authors have reported achieving zero or very low mortality with use of these criteria [444, 445]. Despite the ICG test being associated with some minor, but practical problems, such as the slight invasiveness associated with the injection of ICG and the long time needed for the test, we recommend that the ICG test also be performed in Asia–Pacific countries other than Japan, because the safety of the surgery is coming to be regarded as the first priority in this region.

The MELD score, which is calculated from laboratory values for creatinine, bilirubin, and international normalized ratio for prothrombin time, is well known as a good predictor to guide care in patients with end-stage liver disease awaiting transplantation. However, it is not useful to decide indication of resection, because it assesses only the degree of synthetic dysfunction but not the severity of portal hypertension. The hepatic venous pressure gradient is also a well-known factor adopted in treatment algorithms advocated by the BCLC group. However, it is not used in clinical practice, because of the difficulty of direct measurement.

To increase the safety of surgical resection, it is also important to accurately estimate the liver volume to be resected and the liver volume to be preserved. Recently, a three-dimensional (3-D) virtual hepatectomy simulation software has been developed, which enables estimation of the anatomic relationships between the tumors and vessels in the liver. Preoperative volumetric estimation becomes easier and more accurate with the use of this software [446]. By applying the results of the preoperative volume estimation to Makuuchi’s criteria, the surgical indications in HCC patients with underlying liver cirrhosis can be determined more precisely, increasing the safety of liver surgery. Because this evaluation method requires the aforementioned expensive software and digital data obtained by MDCT, it may be difficult or impossible to apply at all institutions. However, manual volumetric estimation, which was the method employed before the introduction of the 3-D simulation software, is a useful substitute and should be considered in difficult situations. If the liver volume that can be preserved is too small compared with the estimated liver function, portal vein embolization is a good choice to avoid the risk of liver failure. This method, which was originally developed for treatment of hilar bile duct carcinoma [447], can be applied to obtain sufficient remnant liver volume before major hepatectomy for HCC. If preoperative evaluations suggest that the future liver remnant would be insufficient, portal vein embolization is a useful method to ensure the safety of major hepatectomy by increasing the volume of the contralateral “remnant” lobe.

The accumulated experience and tremendous efforts of preceding surgeons have remarkably increased the safety and expanded the indications of liver surgery in patients with HCC. If liver function can be preserved, the range of “technically resectable” HCC will also expand. However, whether HCC tumors are technically/practically resectable or not should be decided considering the clinical practice recommendations at each institution. In addition to the skill level and experience of the surgeons, a multidisciplinary approach is also important to cope with various kinds of complication. Institution-related conditions are expected to become more and more significant in the future.

In conclusion, the resectability of HCC has to be determined with first priority accorded to the safety of resection. Appropriate and accurate preoperative evaluations by expert surgeons and institutions are indispensable.

### The perspective of hepatologists

Hepatic resection is a quite complicated surgical procedure among various operative methods. When considering hepatic resection for HCC, surgeons have to evaluate tumor location and liver functional reserve and decide an appropriate extent of resection and specific resection technique such as limited resection and systematic resection. Although there are several algorithms to guide secure hepatic resection, the detailed operative plan can only be formed by well-experienced hepatobiliary surgeons in marginally resectable cases. Therefore, the role of hepatologists is limited to monitoring surgeons’ skill based on outcomes such as in-hospital mortality. It is well known that in-hospital mortality is strongly affected by the number of hepatic resections performed annually in a hospital [448]. In other words, the resectability of HCC in terms of safety differs markedly among surgeons, hospitals, and countries. Basically, hepatic resection should be performed by surgeons specialized in hepatobiliary surgery rather than general gastroenterological surgeons. If hepatologists judge their surgeons not to be prepared for difficult hepatic surgery, they have to recommend referral to other hospitals with well-experienced hepatobiliary surgeons or, in some “ablatable” cases, to another department with expertise in local ablative therapy.

### Local ablation

#### Recommendations

1. Percutaneous ablation therapies should be performed on patients with HCC, generally for Child–Pugh class A or B patients with three or fewer tumors, each 3 cm or less in diameter (B1).
2. Ethanol injection is a treatment of choice only in cases in which radiofrequency ablation (RFA) cannot be performed safely because of either enterobiliary reflux, adhesion between the tumor and the gastrointestinal tract, or other reasons (B1).
3. RFA is recommended as an image-guided percutaneous ablation technique (A1).
4. RFA is an acceptable alternative to resection for HCC 3 cm or smaller in Child–Pugh class A or B patients (B1).
5. RFA is a first-line treatment in HCC 2 cm or smaller in Child–Pugh class A or B cirrhosis (B1).

Image-guided percutaneous ablation therapies include ethanol injection [449–451], microwave ablation (MWA) [452], radiofrequency ablation (RFA) [453–455], and others. They are potentially curative, minimally invasive, and easily repeatable for recurrence. They are mainly performed on patients with small HCC, generally in Child–Pugh class A or B patients with three or fewer tumors each 3 cm or less in diameter.

Percutaneous ethanol injection was first reported in the early 1980s [449–451], and was long the standard in ablation. Survival of patients treated with ethanol injection has been reported to be 38–60% at 5 years [456–459]. Local tumor progression after percutaneous ethanol injection has been reported to occur in 6–31% depending on the tumor size [456, 458, 460, 461]. Percutaneous ethanol injection has been considered a safe procedure, with mortality and morbidity of 0–3.2% and 0–0.4%, respectively [458–460, 462]. Nowadays, ethanol injection is a treatment of choice only in cases in which RFA cannot be performed safely because of either enterobiliary reflux, adhesion between the tumor and the gastrointestinal tract, or other reasons.

Percutaneous MWA, in which cancer tissue is ablated by dielectric heat produced by microwave energy emitted from the inserted bipolar-type electrode, was introduced into clinical practice in the 1990s [452]. The first-generation MWA has been replaced by RFA in Japan [463], because of small volume of ablation. New-generation MWA systems incorporating antenna cooling and high-power generation have received considerable attention [464]. New-generation MWA may create a more predictable ablation zone, and a larger ablation volume in a shorter procedure time. However, its cumulative reported experience is limited. Further studies are needed, especially from the viewpoint of long-term survival.

In RFA, radiofrequency energy emitted from the exposed portion of the electrode is converted into heat, which causes necrosis of the tumor. RFA has recently been the most widely used ablation technique for HCC. Its survival has been reported to be 39.9–68.5% at 5 years and local tumor progression to be 2.4–27.0% [465–470]. Mortality and morbidity of RFA have been reported to be 0.9–7.9% and 0–1.5%, respectively [465–469]. Compared with RFA alone, combination of RFA with TACE may increase the volume of necrosis [471, 472], and might improve overall survival [473, 474]. Likewise, hepatic arterial balloon occlusion during RFA might extend the area of ablation and decrease tumor recurrence from the same subsegment as the ablated tumor [475].

Irreversible electroporation (IRE) is a nonthermal tumor ablation technique that uses electric pulses to induce cell death, while preserving the structural integrity of bile ducts and vessels. IRE seems to be useful for tumors near a major Glisson’s sheath [476].

There have been five RCTs comparing RFA with ethanol injection. Four of them demonstrated superiority of RFA over ethanol injection, in terms of treatment response, recurrence, and OS [455, 477–479], while the other trial showed that OS was not significantly different between RFA and ethanol injection [480]. Ethanol injection, however, does not require special instruments and is cheaper. Ethanol injection might be a treatment of choice in very small HCC.

An RCT comparing RFA with first-generation MWA demonstrated that the number of treatment sessions was fewer in RFA, although complete therapeutic effect, major complications, and local tumor progression were not statistically different between the two therapies [481].

It is not easy to compare outcomes between RFA and surgical resection; the indications are different between the two treatments. Furthermore, indications for each treatment are different from institution to institution. Thus, a case adjudged to be treatable by RFA or surgical resection at an institution may not be given the same treatment at another. There have been four RCTs comparing RFA with surgical resection. Three of them showed that OS was similar between RFA and surgical resection. A trial on patients with a solitary HCC 5 cm or smaller showed that OS and disease-free survival (DFS) were not statistically different between RFA and resection, but complications were more frequent and severe after surgery [482]. Another trial on patients with nodular diameters of less than 4 cm and up to 2 nodules showed that there were no statistically significant differences between RFA and surgical resection in terms of OS and RFS [483]. In another trial on patients with HCC 3 cm or smaller in diameter, there was no significant difference in DFS or OS between RFA and hepatectomy, although the incidence of postoperative complications and hospital stay were significantly greater in hepatectomy [484]. The remaining study on patients within the Milan criteria showed that OS and RFS were significantly lower in RFA than in surgical resection [485].

Concerning OS, some nonrandomized comparative studies reported that RFA had similar survival to resection [486–497], while others found that resection was associated with higher survival [426, 498–502]. Even in studies which reported that surgical resection was superior to RFA, there was no significant difference in OS between RFA and surgical resection in patients with HCC 2 cm or smaller in diameter [426] or 3 cm or smaller in diameter [499–501]. In one study, RFA had better long-term survival than surgical resection after propensity score analysis [503]. RFA was associated with fewer major complications [494, 500] and shorter hospital stay [494]. RFA may be more cost-effective than surgical resection [504]. Most studies reported that RFS was higher in surgical resection than in RFA, although OS was not significantly different between RFA and surgical resection in them. This is probably because surgical resection removes a much larger volume of liver parenchyma, which may result in removal of some occult metastases and reduction of new carcinogenesis but may be prone to liver decompensation. In addition, most recurrence can be treated curatively by iterative RFA [469] but not by repeated surgical resection. Although further RCTs are warranted to compare ablation with surgical resection [505], data available at present suggest that OS is not significantly different between RFA and surgical resection. Various innovations, such as CEUS [506] and multimodality fusion imaging [507], would improve outcomes in ablation.

Ablation is less invasive and less expensive. Because patients with HCC have been markedly aging, minimally invasive therapies such as ablation would play a more important role. Because many Asian countries are still developing, from the viewpoint of medical economics, highly cost-effective therapies such as ablation should have priority. Ablation techniques, especially RFA, may be an alternative to surgery in selected cases.

### Transarterial chemoembolization (TACE)

#### Recommendations

1. Transarterial chemoembolization (TACE) is recommended as a first-line treatment of HCC for patients with unresectable, large/multifocal HCCs who do not have vascular invasion or extrahepatic spread (A1).
2. Selective TACE can be performed in patients with small tumors in whom ablation is difficult to perform because of tumor location or medical comorbidities (B1).
3. Selective or superselective TACE should be attempted in order to preserve nontumorous liver parenchyma, maximize treatment effect, and minimize complications (A1).
4. TACE using drug-eluting beads has similar therapeutic efficacy with less systemic adverse events compared with conventional TACE (B2).
5. Other treatment strategies might be considered for patients with HCC who are not suitable for or do not response to repeated TACE (B2).
6. Transarterial radioembolization (TARE) with yttrium-90-loaded resin/glass beads may be used as an alternative locoregional treatment for unresectable HCC (B2).

Although the normal liver receives a dual blood supply from the hepatic artery and the portal vein, HCC is supplied almost exclusively by the hepatic artery [508]. TACE exploits the preferential hepatic arterial supply of HCC for targeted delivery of chemotherapeutic agents, usually mixed with lipiodol, followed by embolization or reduction in arterial flow using various types of particles (e.g., gelfoam particles), while sparing the surrounding liver parenchyma [509]. TACE is currently considered as the mainstay of therapy for unresectable, large/multifocal HCCs without vascular invasion or extrahepatic spread [510]. TACE provided a significant survival benefit in selected HCC patients with preserved liver function and adequate performance status [511–514]. Therefore, the guidelines published by the EASL and AASLD recommend TACE as a first-line, noncurative therapy for nonsurgical patients with large/multifocal HCC who do not have vascular invasion or extrahepatic spread [2, 63]. In addition, according to the guidelines published by the JSH [515], hepatectomy or TACE is recommended if there are 2 or 3 tumors of less than 3 cm, and TACE or hepatic arterial infusion chemotherapy is recommended if there are 4 or more tumors. In addition, TACE can be performed in patients at early stage in whom RFA is difficult to perform because of tumor location or medical comorbidities [516]. TACE is also the first-line therapy for downstaging tumors that exceed the criteria for LT.

As TACE usually does not induce significant liver dysfunction even in cirrhotic patients and treatment-related mortality is less than 5% [516, 517], the benefits of TACE procedure should not be offset by treatment-induced liver failure. TACE is associated with transient postembolization syndrome, but incidence of severe events has been reported to be less than 5%, including hepatic insufficiency, liver abscess, acute cholecystitis or gastrointestinal bleeding [517, 518]. Important predisposing factors are major portal vein obstruction, compromised hepatic functional reserve, biliary obstruction, previous biliary surgery, excessive amount of iodized oil, and nonselective embolization [519]. Therefore, selective or superselective TACE should be attempted to maximize tumor necrosis and to minimize procedure-related complications by preserving nontumorous liver parenchyma [520, 521].

However, there is no standardized protocol for TACE in terms of treatment schedule or type and dosage of anticancer agent. In addition, predictions of its therapeutic efficacy are limited by the use of nonstandardized embolic material. TACE performed with drug-eluting beads (DEB-TACE) loaded with doxorubicin has been shown to modify the pharmacokinetics of the injected chemotherapy, allowing longer intratumoral exposure and less systemic exposure of the drug, reducing toxicity [522, 523]. In prospective clinical trials, liver toxicity and systemic adverse effects occur less frequently after DEB-TACE than conventional TACE. Although there is no significant benefit of DEB-TACE over conventional TACE with respect to objective response, selected patient groups such as those with Child–Pugh class B, ECOG performance status 1, bilobar disease, and recurrent disease showed a significant increase in objective response in DEB-TACE group [524]. Furthermore, DEB-TACE was associated with improved tolerability, with a significant reduction in serious liver toxicity and side-effects [524]. Despite these promising results, use of DEB-TACE in Asia has been relatively low compared with Western countries [2, 415, 525]. So far, in Asia, there has been no robust evidence favoring use of DEB-TACE in terms of efficacy and cost-effectiveness. Therefore, further research is required to address this issue.

Although TACE is considered the standard of care for nonsurgical HCCs that are also ineligible for percutaneous ablation, those with so-called bulky tumor burden (tumor size >5 cm) and Child–Pugh class B showed the worst survival outcomes (median OS of about 9 months) [526]. Furthermore, several scoring systems [i.e., SNACOR [527], hepatoma-embolisation prognostic (HAP) score [528], modified HAP score [529], Selection for TrAnsarterial chemoembolisation TrEatment (STATE) score [530], and the Chiba HCC in intermediate-stage prognostic (CHIP) score [531]] have been developed, identifying a subgroup with unfavorable outcomes. Among them, STATE score based upon tumor burden (up-to-7 criteria), albumin level, and C-reactive protein level was suggested as an objective point score to guide the decision regarding the first treatment, showing that lower STATE score was associated with worse outcome [530]. In a similar context, the Assessment for Retreatment with TACE (ART) score, an objective point score to guide the decision regarding retreatment with TACE, was developed based upon an increase of aspartate aminotransferase by >25%, Child–Pugh score increase, and absence of radiological response [532]. Higher ART score was associated with major adverse events after the second TACE (*P* = 0.011) [532]. Based upon these findings, sequential use of the STATE and ART scores was suggested to identify the most suitable and unsuitable patients for multiple TACE sessions [530]. So, for such a population with relatively unfavorable outcomes primarily owing to tumor burden and/or liver function, other treatment options based upon multidisciplinary approaches, including a switch of treatment modality from TACE to sorafenib or hepatic arterial infusion chemotherapy, might also be considered. Vice versa, even for large/multinodular HCC, active curative treatments including LT (e.g., within up-to-7 criteria), or so-called downstaging strategies might be tried in selected cases [533–535].

According to conventional size-based response evaluation criteria, i.e., World Health Organization (WHO) criteria [536], the reported rate of objective response ranges between 16 and 60% [513, 517]. The Response Evaluation Criteria in Solid Tumors (RECIST) [537] generally ignore tumor necrosis, and thus may underestimate treatment response [538]. In contrast, two enhancement criteria, the EASL criteria [539] and the modified RECIST (mRECIST) [540], have demonstrated superior efficacy for assessing treatment response and predicting survival outcome compared with the WHO criteria or RECIST [541–543]. The objective response rate using enhancement criteria ranges between 58 and 86%, and 20–41% achieve complete response [538, 541–543]. In addition, the biological response based upon changes in tumor markers after treatment might be used as an ancillary method for assessment of overall response [544, 545].

Another issue related to TACE is the concept of “failure” or “refractoriness” to TACE. So far, several studies have tried to address this [366, 525, 546–548]. The JSH has provided a definition of TACE failure/refractoriness as two or more consecutive ineffective responses seen within the treated tumors, two or more consecutive progressions in the liver (including an increase in the tumor number), continuous elevation of tumor markers right after TACE, appearance of vascular invasion, and appearance of extrahepatic spread [366]. Similarly, according to Raoul et al. [546], a switch of treatment modality from TACE to others including sorafenib might be considered for those who have progression after two sessions of TACE. However, there is still no consensus regarding the definition of TACE failure or refractoriness. Moreover, there is no proven therapy for the purpose of rescue, although sorafenib rescue might improve survival in patients who experience TACE failure, compared with those who continue TACE [549, 550]. Other treatment modalities including internal or external radiotherapy and new molecular targeted agents have been studied as potential rescue therapies for patients with TACE failure.

Many attempts have been made to improve the treatment outcomes of TACE. Combination of sorafenib and TACE might be an eligible option [551]. However, a RCT comparing the efficacy in HCC treated with sorafenib or placebo plus DEB-TACE showed that combination therapy did not improve outcome [552].

Transarterial radioembolization (TARE) involves injection of implantable radioactive microspheres into tumor-feeding arteries in order to expose the tumor to highly concentrated radiation while protecting the normal parenchyma. TARE using yttrium-90 is an evolving and promising regional therapy, which can complement or replace TACE [2, 415, 553]. In a European phase II study of patients with intermediate or advanced HCC, TARE resulted in 40.4% objective tumor response rate with median survival of 15 months [554]. In another large retrospective cohort study conducted in the USA, the median survival of TARE-treated patients with portal vein invasion was significantly shorter than those without invasion (10 versus 15.3 months) [555, 556]. In a recent prospective multicenter Korean study, the 3-month tumor response rate was 57.5% and the 3-year OS rate was 75% [557]. Although there is not enough evidence confirming clinical benefit of TARE compared with conventional TACE, TARE might be recommended to patients who are not good candidates for TACE due to bulky tumor and/or portal vein invasion, based on published data.

### Radiation therapy

#### Recommendations

1. Although stereotactic body radiotherapy (SBRT) and proton beam (also carbon ion beam) are reasonable options for patients who have failed other local therapies, radiotherapy (RT) has not been shown to improve outcomes for patients with HCC. However, RT may be considered for symptomatic bony metastases (C2).

Although HCC is considered to be a radiosensitive tumor, it also is located in a radiosensitive organ. Due to the development of three-dimensional conformal radiation therapy (3D-CRT), radiotherapy (RT) can be performed more safely for patients with HCC without severe toxicity. Technological developments for targeting HCC precisely with RT [intensity-modulated RT (IMRT) and image-guided approaches, including stereotactic body radiotherapy (SBRT)] can improve the benefit and reduce the risk. However, they do not alter the high recurrence rates in other nontreated areas of the liver. There are no large-scale RCTs demonstrating an effect of any form of RT on survival and no consensus regarding the optimal use of this therapy. Thus, RT is not recommended in the AASLD and EASL guidelines for treating HCC [2, 63]. Even though strong evidence is lacking, RT may be one of the promising treatment options for HCC.

### Indications

The lack of strong evidence to support RT for patients with HCC is reflected in the various recommendations of expert groups in different countries. The AASLD and EASL guidelines do not address use of external-beam RT for treatment of HCC [2, 63]. Consensus-based guidelines from the National Comprehensive Cancer Network (NCCN) list external-beam RT (conformal or stereotactic) as an alternative option to ablation or arterially directed therapies for patients with unresectable HCC who have contraindications for liver transplantation. An expert consensus group of the Americas Hepato-Pancreato-Biliary Association (AHPBA) concluded that RT can provide local control for some unresectable HCC lesions, that better RT planning and delivery (for example, hypofractionation, stereotactic treatment, proton beam, and carbon ion beam therapy) have the advantage of increasing the radiation dose to unresectable HCC without causing severe toxicity, and that strategies combining RT with other therapies merit continued evaluation [558]. SBRT and proton beam (also carbon ion beam) are reasonable options for patients who have not responded to other local modalities and have no extrahepatic disease, limited tumor burden, and relatively good liver function. Where available, proton beam and carbon ion beam irradiation is a reasonable approach for patients with large HCC with or without tumor thrombus of vessels.

### Contraindications

The radiation dose must be relatively low to minimize the radiation effect on normal liver included in the treatment field. Use of RT should be limited to patients with sufficient liver function (Child–Pugh score 7 or less) and liver volume outside the radiation field. Patients with Child–Pugh score of 8 or more have elevated risk of radiation-induced hepatic toxicity or liver failure [559]. A relative contraindication to RT is previous hepatic radiation to the same segment of the liver. While retreatment may be possible in select cases, these patients should be evaluated in a tertiary care center by experts on hepatic RT [560, 561].

### Efficacy

#### 3D-CRT

With the development of 3D-CRT techniques, RT can be performed more safely to the HCC with less liver toxicity. Most available data are from retrospective or single-center studies [562, 563]. A phase II trial in France reported sustained local tumor control in 78% of patients with early-stage HCC (one nodule and ≤5 cm, or two nodules and ≤3 cm) who were treated with 3D-CRT [564]. One of the problems with RT is the high intrahepatic recurrence rate outside of the high-dose irradiation area, which may be caused in part by difficulties with accurately targeting HCC during conventional RT treatment planning [565].

#### SBRT

SBRT (sometimes called stereotactic radiosurgery) is a technique in which a limited number of high-dose RT with hypofractionation (typically 3–6) are delivered to a small, definite target using multiple, nonparallel radiation beams. The beams converge on the target lesion, minimizing radiation exposure to other normal tissue or organs. This targeting makes it possible to treat a lesion in either a single or limited number of dose fractions. Experience with SBRT for HCC is increasing [566–569]. In the largest series, 93 patients (Child–Pugh A: 69 patients; Child–Pugh B: 24 patients) with small HCCs (median 2 cm; range 1–6 cm) who were not eligible for surgical resection or RFA were treated with SBRT [566]. The in-field complete response rate was 16%, but the in-field progression-free survival at 3 years was 92%.

#### Charged-particle radiation therapy

There is a growing body of evidence, primarily from Japan, supporting use of proton beam and carbon ion beam irradiation, particularly for patients with large tumors or portal vein thrombus [570–573]. In one study, 162 patients with 192 HCCs were treated with proton beam irradiation [570]. Most tumors had diameter of 3–5 cm. The majority of the patients had past history of receiving other nonsurgical treatments. The 5-year local control and 5-year survival rates were 87 and 24%, respectively.

### Complications

Minimizing radiation-induced complications depends on careful patient selection and radiation treatment planning. The most common acute side-effects include transient fatigue, nausea, vomiting, and right upper quadrant pain. Possible long-term side-effects include worsening hepatic function with ascites, edema, hepatomegaly, thrombocytopenia, and elevated liver function tests. Rarely, cases of radiation-induced biliary stenosis, portal vein thrombosis, or death from radiation-induced liver failure have been reported [574].

### Response assessment

Dynamic CT or dynamic MRI is usually performed at 4 weeks, 2–3 months later, and then at 3-month intervals for at least 1 year. If there has been no recurrence of disease after a year, imaging will be performed every 4–6 months.

### Systemic therapy

#### Recommendations

1. Sorafenib is recommended for the first-line treatment of advanced-stage patients (macrovascular invasion or extrahepatic metastasis) who are not suitable for locoregional therapy and who have Child–Pugh class A liver function (A1).
2. Sorafenib may be used with caution in patients with Child–Pugh class B liver function (B2).

### Sorafenib

Sorafenib, a multikinase inhibitor of Raf, vascular endothelial growth factor receptor (VEGFR), platelet-derived growth factor receptor (PDGFR), c-kit, Flt-3, and RET [575], has been approved for the treatment of advanced HCC in patients with Child–Pugh class A liver function worldwide. The approval is generally based on the results of two phase III, double-blind, placebo-controlled trials [576, 577]. The first trial (SHARP trial) was conducted primarily in Europe and the USA (HCV: 28.1%, alcoholic liver disease: 26.4%) with the primary end point of OS. The second trial was conducted primarily in the Asia–Pacific population (HBV: 73%) with an almost identical design to the SHARP trial. Sorafenib resulted in a similar survival benefit in these two different patient populations. The hazard ratios of OS and time to radiological progression were 0.69 and 0.58 in the SHARP trial and 0.68 and 0.57 in the Asia–Pacific trial. Exploratory subgroup analyses of the two trials indicated that sorafenib treatment prolonged survival regardless of patient age, performance status, and tumor burden (vascular invasion or extrahepatic spread). However, sorafenib rarely induced radiological responses (SHARP trial: 2%, Asia–Pacific trial: 3.3%). Sorafenib at dosage of 400 mg twice daily is generally well tolerated. The most common drug-related adverse events included diarrhea, fatigue, hand–foot skin reaction, and rash/desquamation, most of which were grade 1 or 2. The most common causes of treatment interruption or dose reduction were hand–foot skin reaction, rash, and diarrhea.

The efficacy of sorafenib in patients with Child–Pugh class B liver function has never been prospectively studied by RCTs. Several noninterventional studies investigated the efficacy and safety of sorafenib in HCC patients with Child–Pugh class A versus class B liver function. HCC patients with Child–Pugh class B liver function, compared with those with Child–Pugh class A liver function, had shorter duration of sorafenib use (Child–Pugh class B: 8.4 weeks; Child–Pugh class A: 13.6 weeks) [578] and shorter median OS (Child–Pugh class B: 3.8–4.5 months; Child–Pugh class A: 10–13 months) [579–581] but similar rates of adverse events. Patients with Child–Pugh score 7, compared with patients with Child–Pugh score 8 or 9, had higher median OS time, but the difference did not reach statistical significance [580–583]. Taken together, patients with Child–Pugh class B liver function did not suffer excessive risk with sorafenib use, but they were more likely to develop hepatic decompensation [584], which limited continuation of sorafenib and thus survival. Therefore, sorafenib may be used with caution in patients with Child–Pugh score 7 and is generally not suggested for patients with Child–Pugh score >7 or decompensated cirrhosis.

### Regorafenib

Regorafenib, a novel multikinase inhibitor, has more potent inhibitory activities against multiple angiogenic pathways (VEGFR, PDGFR, TIE2, and FGFR) and oncogenic pathways (RET, KIT, c-RAF/RAF-1, and BRAF) than sorafenib [585]. Regorafenib, administered at 160 mg once daily for 3 weeks in each 4-week cycle, has been investigated for its efficacy and safety as a second-line treatment in a phase III double-blind RCT (RESORCE trial) [586]. Regorafenib, compared with placebo, significantly reduced the risks of death (HR 0.62; 95% CI 0.50–0.78; *p* < 0.001) and progression or death (HR 0.46; 95% CI 0.37–0.56; *p* < 0.001) in 573 HCC patients (regorafenib: 379 patients; placebo: 194 patients) with Child–Pugh class A liver function who had progression on sorafenib. The median OS and progression-free survival (regorafenib versus placebo) were 10.6 versus 7.8 months and 3.1 versus 1.5 months, respectively. The overall response rate (regorafenib versus placebo) was 10.6 versus 4.1%, respectively (*p* = 0.005). Rates of grade ≥3 adverse events were 79.7% with regorafenib and 58.5% with placebo. The most common grade ≥3 adverse events included hypertension, hand–foot skin reaction, fatigue, and diarrhea.

### Treatment algorithm

**Fig. 2:**
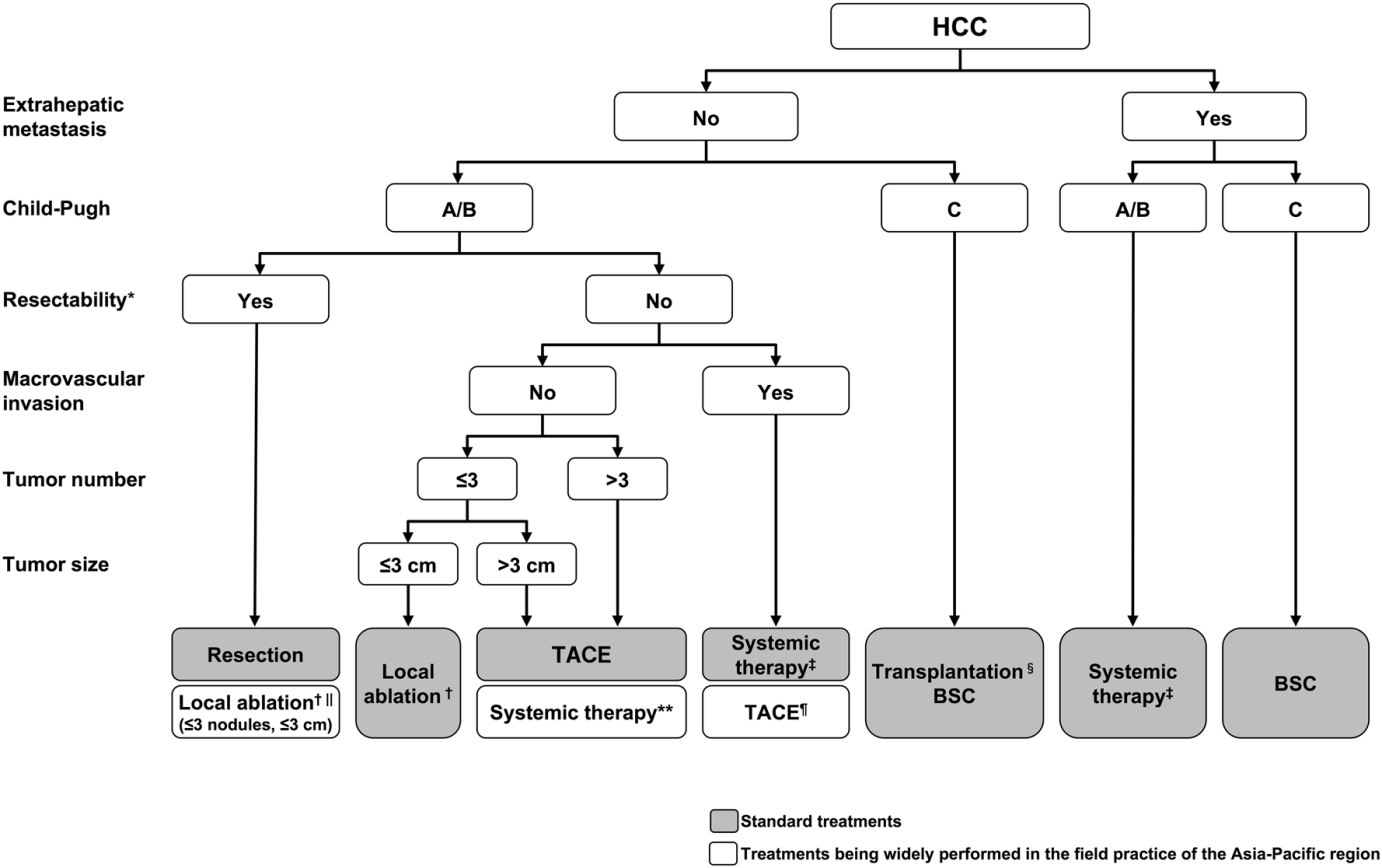
Treatment algorithm for hepatocellular carcinoma (APASL 2016). *Decisions regarding resectability should be discussed in a multidisciplinary team. †RFA is recommended as the first choice for the local ablation. ‡Currently, sorafenib and regorafenib are drugs that have shown clinical benefits in phase III studies. See text for use of systemic therapy. §Liver transplantation is recommended when indicated. ||Local ablation is an alternative treatment in resectable patients (≤3 cm and ≤3 nodules). Choice of treatments should be discussed in a multidisciplinary team. ¶TACE is an alternative treatment in patients with macrovascular invasion (no extrahepatic metastasis). Choice of treatments should be discussed in a multidisciplinary team. **Treatment conversion from TACE to systemic therapy is recommended for patients in whom TACE is expected to be ineffective

The latest treatment algorithm is evidence based and attempts to be comprehensible and suitable for universal use in the Asia–Pacific region, which has a diversity of medical environments (Fig. 2). The order of columns corresponds to the decision-making process of treatment in field practice. Standard treatments with high evidence levels and treatments being widely performed in field practice in the Asia–Pacific region are demonstrated. The results of ongoing trials, which will be announced in the near future, or further planning of prospective studies will present possibilities for changing standard treatments. It is greatly hoped that promising results will be delivered from the Asia–Pacific region. Although there are various treatments being performed in limited institutions or countries, those treatments which do not have sufficient supporting evidence are not indicated in terms of universal use in this region.

RFA for resectable patients (≤3 cm, ≤3 nodules) and TACE for patients with macrovascular invasion (no extrahepatic metastasis) are often performed in field practice in the Asia–Pacific region. The JSH consensus-based guidelines and the Hong Kong Liver Cancer staging system are similar protocols recommended based on these points [414, 548]. Despite insufficient evidence for standard treatments at the moment, RFA for resectable patients (≤3 cm, ≤3 nodules) and TACE for patients with macrovascular invasion (no extrahepatic metastasis) are categorized as treatments being widely performed in field practice in the Asia–Pacific region.

Recently, the concept of conversion from TACE to sorafenib before the appearance of macrovascular invasion or extrahepatic metastasis has been advocated by clinicians from both Europe and Japan [366, 546, 548]. This point of controversy in clinical practice has been discussed since the approval of sorafenib for treatment of HCC. Although only a few retrospective studies have reported the effectiveness of this concept [549, 550, 587], conversion from TACE to systemic therapy appears to be a reasonable treatment strategy. In fact, in field practice, sorafenib has been administered to a considerable number of patients without either macrovascular invasion or extrahepatic metastasis [588]. Thus, this treatment algorithm recommends treatment conversion from TACE to systemic therapy for patients in whom TACE is ineffective.

The other unique point of this algorithm is the indication for hepatic resection. It does not include strictly defined conditions for hepatic resection. According to the Japanese guidelines, resection is recommended in several treatment arms, thereby making these recommendations complicated and confusing [366, 547]. On the other hand, indications for resection are limited, such as a single lesion and normal hepatic portal vein pressure, according to the BCLC staging system [409]. These selection criteria appear to be too strict and unsuitable for use in the Asia–Pacific region. It may be difficult to define criterion for resectability that are generally applicable in countries with varying medical environments. In this treatment algorithm, indications for resection are not strictly defined in order to allow surgeons and hepatologists to collaborate in deciding on therapeutic strategies. Thus, this algorithm recommends that decisions regarding resectability are discussed by a multidisciplinary team, including surgeons and hepatologists. It is also important for surgeons and hepatologists to provide feedback on treatment outcomes to one another.

## Clinical trials for new compounds on the horizon

### Molecular targeted agents

In addition to sorafenib and regorafenib, a variety of molecular targeted agents have been thoroughly investigated, including sunitinib [589], brivanib [590, 591], linifanib [592], ramucirumab (angiogenesis inhibitors) [593], erlotinib (EGFR inhibitor) [594], and everolimus (mTOR inhibitor) [595]. However, none have shown survival benefits in either first-line or second-line setting in phase III RCTs. The results of large randomized phase III trials for lenvatinib (first-line) and cabozantinib (second-line) will soon be available. All of these trials were conducted in biomarker-unselected HCC patients. Recently, tivantinib was found effective in MET-high subgroup of patients [596], and MET-high-enriched randomized phase III studies were initiated. The latest early-phase trials for selective c-Met inhibitors, tepotinib [597], capmatinib [598], as well as selective FGFR4 inhibitor FGF401 are being conducted in biomarker-enriched HCC patients.

### Immunotherapy

Immuno-oncology is an emerging area of drug development. Major breakthroughs have been achieved in agents targeting immune checkpoint proteins, such as cytotoxic T lymphocyte antigen-4 (CTLA-4) and programmed cell death-1 (PD-1), in patients with various types of cancer [599–603]. These immune checkpoint inhibitors restore and sustain activation of either primed or effector T cells to exert T cell-mediated cancer cell killing. Tremelimumab, an anti-CTLA-4 antibody, resulted in an objective response rate of 17.6%, a disease control rate of 76.4%, and time to progression of 6.48 months in 21 HCV-related HCC patients who had failed at least one line of systemic therapy [604]. Nivolumab, an anti-PD-1 antibody, is currently being investigated as a second-line therapy in a phase I/II trial. The preliminary data from its dose expansion cohort showed an objective response of 20% and a 9-month OS rate of 74% in 214 HCC patients [605]. The objective responses were observed in all etiology groups. Adverse events were generally tolerable and manageable in these two trials. Moreover, a significant proportion of patients with chronic HBV or HCV infection had reduction of viral load with study treatment. These promising results indicate an important step toward a new paradigm of systemic therapy for advanced HCC. More clinical trials using immune checkpoint inhibitors alone or in combination with immunotherapy or molecular targeted therapy are ongoing (Table 5).

**Table 5:** Ongoing clinical trials of immune checkpoint inhibitors for advanced hepatocellular carcinoma

Drug	Phase	Design	Study number
*First*-*line*			
Nivolumab versus sorafenib	III	Randomized, open label	NCT02576509
Nivolumab plus ipilimumab (anti-CTLA-4) versus nivolumab versus sorafenib	II	Randomized, open label	NCT01658878
*Second*-*line*			
Nivolumab	Ib	Open label	NCT01658878
Pembrolizumab (anti-PD-1)	II	Open label	NCT02702414
Pembrolizumab versus placebo	III	Randomized, double blind, placebo controlled	NCT02702401
Nivolumab plus galunisertib (GSK-β inhibitor)	II	Open label	NCT02423343
Durvalumab (anti-PD-L1) plus tremelimumab versus durvalumab versus tremelimumab	II	Randomized, open label	NCT02519348
Durvalumab plus ramucirumab (anti-VEGFR)	I	Open label	NCT02572687
PDR001 (anti-PD-1) plus capmatinib (cMet inhibitor) versus PDR001	Ib/II	Open label	NCT02795429
